# Discovery of a Phenylalanine‐Derived Natural Compound as a Potential Dual Inhibitor of MDM2 and MDMX

**DOI:** 10.1002/cmdc.202500397

**Published:** 2025-07-22

**Authors:** Ja Young Cho, Sanghwa Park, Taejung Kim, Junghye Eom, Jung‐Rae Rho, Hyoung‐Woo Bai

**Affiliations:** ^1^ Prokaryote Research Division Nakdonggang National Institute of Biological Resources (NNIBR) Sangju 37242 Republic of Korea; ^2^ Bio‐resources Bank Division Nakdonggang National Institute of Biological Resources (NNIBR) Sangju 37242 Republic of Korea; ^3^ Institute of Natural Products Korea Institute of Science and Technology (KIST) Gangneung 25451 Republic of Korea; ^4^ Department of Oceanography Kunsan National University Gunsan 54150 Republic of Korea; ^5^ Research division for Biotechnology Advanced Radiation Technology Institute (ARTI) Korea Atomic Energy Research Institute (KAERI) Jeongeup 56212 Republic of Korea

**Keywords:** MDM2/MDMX dual inhibition, *Micromonospora*, novel phenylalanine derivatives, novel small molecules, protein–protein interaction inhibitor

## Abstract

Dual inhibition of the negative p53 regulators MDM2 and MDMX has emerged as an effective strategy in p53‐based anticancer therapy. However, dual inhibitors are limited, and many inhibitors exhibit poor pharmacokinetic properties and fast dissociation kinetics. Among newly identified microbial metabolites, the novel phenylalanine‐derived compound P5 isolated from *Micromonospora* sp. MS‐62 (FBCC‐B8445) exhibits inhibitory activity against both MDM2 and MDMX. The binding of P5 to MDM2 and MDMX is demonstrated by surface plasmon resonance, which reveals nanomolar‐level affinity and slow dissociation kinetics (KD = 46 nM for MDM2; 576 nM for MDMX). This dual inhibitory activity was further supported by molecular docking, which reveals binding of P5 to the p53‐binding pockets of both MDM2 and MDMX through extensive noncovalent interactions. In cell‐based assays, P5 reduced cancer cell viability across several human cell lines. Furthermore, *in silico* analysis indicates favorable pharmacokinetic properties, including gastrointestinal absorption, blood–brain barrier permeability, and compliance with Lipinski's and Veber's criteria. P5 combines dual‐target engagement with binding persistence and favorable pharmacokinetic characteristics, addressing limitations of earlier inhibitors. P5 is a potential lead compound for the development of MDM2/MDMX‐targeted anticancer agents.

## Graphical abstract

P5, a novel phenylalanine‐derived compound isolated from *Micromonospora* sp. MS‐62, exhibits dual inhibition of MDM2 and MDMX. Molecular docking and SPR confirm its nanomolar affinity and sustained binding kinetics. In silico profiling reveals high GI absorption, BBB permeability, and drug‐likeness, highlighting its potential as a lead for p53‐targeted cancer therapy.

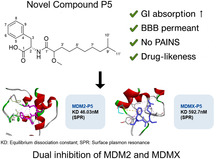

## 1 Introduction

Decades of extensive and innovative research have been conducted in cancer treatment. Nevertheless, it remains challenging in modern medicine, largely due to limitations in clinical application.^[^ ^1^ ^]^ Moreover, the number of deaths caused by cancer continues to rise annually.^[^ ^2^ ^]^ Targeting tumor‐specific molecular drivers has emerged as a critical strategy to address these limitations.^[^ ^2^, ^3^ ^]^ MDM2 and MDMX are well‐established pharmacological targets due to their role as negative regulators of the tumor suppressor p53.^[^ ^4^, ^5^, ^6^ ^]^ These proteins suppress p53 by binding a short α‐helix in its N‐terminal transactivation domain through conserved hydrophobic pockets that are structurally distinct from their catalytic regions.^[^ ^4^, ^5^, ^6^ ^]^ This binding interface is well characterized and widely applied in the development of small molecule inhibitors that disrupt protein–protein interactions.^[^ ^4^, ^5^, ^6^ ^]^ p53 regulates apoptosis and cell cycle arrest,^[^ ^4^, ^5^, ^6^ ^]^ and its dysfunction, observed in over 50% of human cancers, arises from either mutation or regulatory suppression.^[^ ^4^, ^5^ ^]^ In cancers with wild‐type p53, overexpression of MDM2 or MDMX suppresses p53 activity, making them key targets for its reactivation.^[^ ^4^, ^5^, ^6^ ^]^

**Figure 1:**
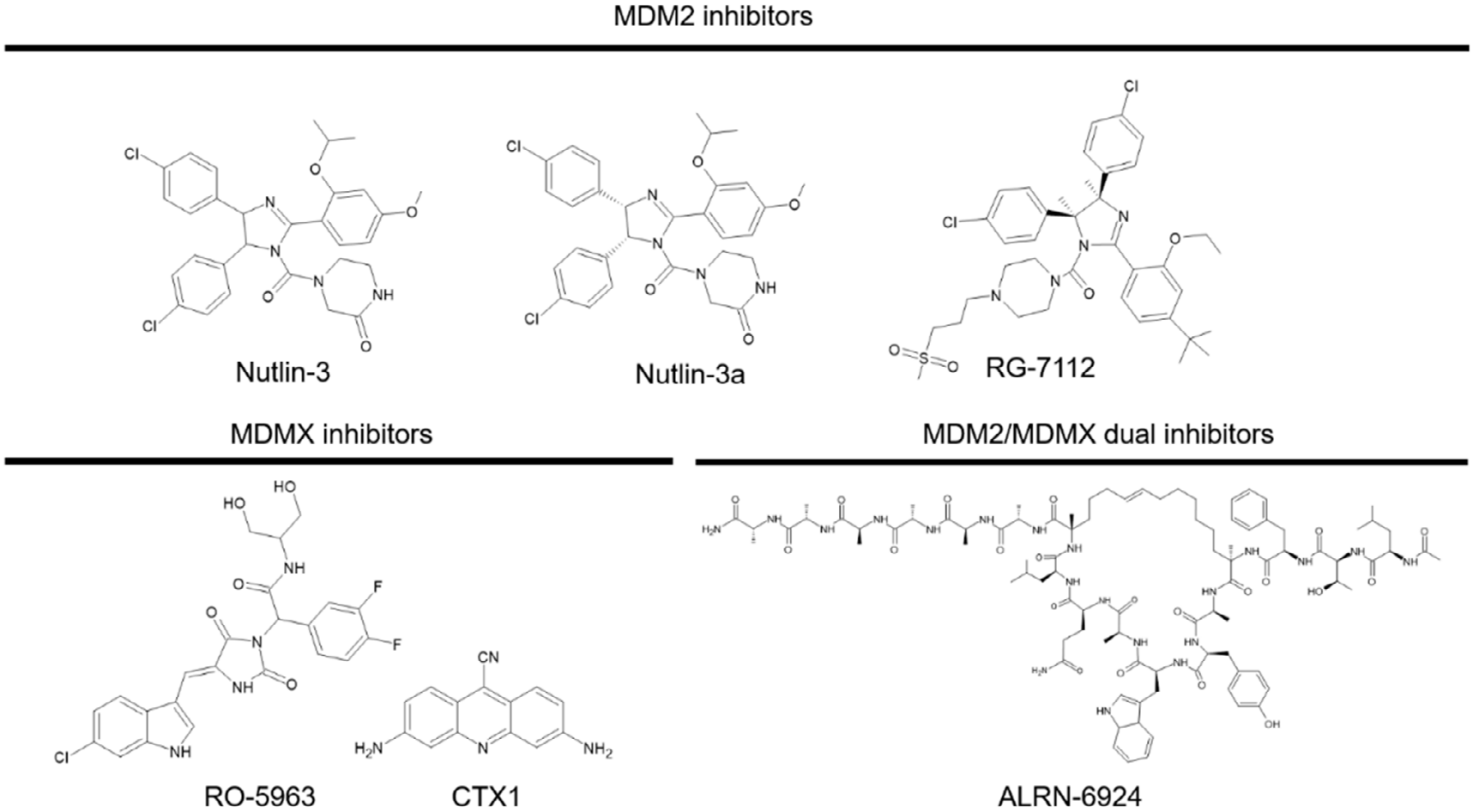
Representative MDM2 (nutlin‐3, nutlin‐3a, and RG‐7112), MDMX (RO‐5963, CTX1), and MDM2/MDMX dual inhibitors (ALRN‐6924).

However, most small molecule inhibitors developed to date, including Nutlin‐3 and derivatives, selectively target MDM2 and do not bind MDMX.^[^ ^4^ ^]^ This limited specificity often results in incomplete p53 pathway reactivation and therapeutic resistance, particularly in tumors with elevated MDMX expression.^[^ ^4^ ^]^ In addition, many of these compounds exhibit suboptimal pharmacokinetic properties, and some exhibit rapid dissociation from the target, both of which have hindered clinical translation.^[^ ^7^, ^8^ ^]^ These limitations have been addressed by ALRN‐6924, a stapled α‐helical peptide that simultaneously targets both MDM2 and MDMX. It exhibits improved pharmacokinetic properties and slower dissociation kinetics compared to small molecule inhibitors.^[^ ^4^, ^5^ ^]^ ALRN‐6924 is currently in Phase II clinical trials, providing clinical support for the dual inhibition strategy. **Figure** **1** presents representative structures of known MDM2 and MDMX inhibitors, including the MDM2‐selective small molecules Nutlin‐3, Nutlin‐3a, and RG‐7112; the MDMX‐selective compounds RO‐5963 and CTX1; and the dual MDM2/MDMX inhibitor ALRN‐6924. These examples illustrate the diversity of molecular scaffolds targeting the p53–MDM2/MDMX interaction.

Consequently, the development of new chemical scaffolds capable of dual MDM2/MDMX inhibition with improved drug‐like characteristics remains a key objective in p53‐targeted therapy.^[^ ^4^, ^5^ ^]^ Natural compounds derived from microbial sources have already contributed to FDA‐approved drugs by modulating molecular targets related to tumor growth, survival, and apoptosis.^[^ ^9^ ^]^ These compounds not only provide validated bioactivity but also offer structurally diverse scaffolds suitable for targeting protein–protein interactions. Therefore, exploring unexplored microbial resources represents a rational strategy for identifying novel dual inhibitors.^[^ ^10^, ^11^, ^12^ ^]^

The objective of this study was to identify a novel dual MDM2/MDMX inhibitor with improved pharmacokinetic characteristics by exploring microbial natural products. Five phenylalanine‐derived compounds were isolated from a *Micromonospora* sp. MS‐62 strain associated with freshwater sponges, and their structures were elucidated using mass spectrometry, nuclear magnetic resonance (NMR) spectroscopy, and circular dichroism (CD) spectroscopy. One compound, designated P5, was selected for further investigation. Its interaction with the p53‐binding pockets of MDM2 and MDMX was assessed through molecular docking and validated by surface plasmon resonance (SPR). Cell‐based assays were conducted to evaluate its effects on cancer cell viability, and pharmacokinetic properties were evaluated using advanced in silico prediction models.

## 2 Results and Discussion

### 2.1 Bacterial Strain Isolation from Freshwater Sponge

**Figure 2:**
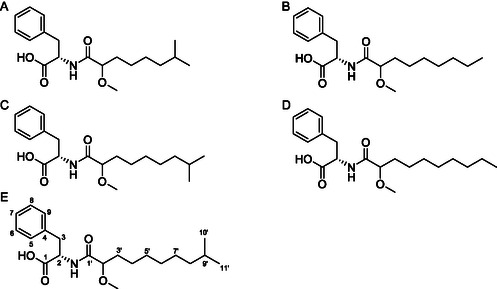
A–E) Structures of novel phenylalanine derivatives P1–P5. P5 contains the core scaffold with numbered carbon atoms.

Between 1998 and 2017, a total of 774 novel bioactive compounds were reported from marine sponge‐associated microorganisms, many of which were identified as small molecule inhibitors targeting proteins relevant to cancer therapy.^[^ ^2^, ^10^, ^11^, ^13^ ^]^ By comparison, microorganisms associated with freshwater sponges remained largely unexplored during this period, despite their potential to yield structurally unique secondary metabolites. To investigate this underexplored niche, a freshwater sponge was collected from the Geumgang River in Muju, Jeollabuk‐do, South Korea. Based on spicule morphology (Figure 1, Supporting Information) and cytochrome oxidase subunit I (Co1) gene sequencing, the sponge was identified as *Spongilla lacustris* (Figure 2, Supporting Information). After 7 days of cultivation of homogenized sponge tissue, microbial isolates were obtained. Among them, seven *Micromonospora* strains were identified through 16S rRNA gene sequencing.

The isolates were cultured in Reasoner's 2 A (R2A) medium for 14 days, and crude extracts were prepared by sequential liquid–liquid extraction with dichloromethane, 85% methanol, and hexane. Given that microbial secondary metabolites are often enriched in organic solvent fractions, only the methanol extracts were screened for biological activity against PC‐3 prostate cancer cells. Cell viability was assessed at a concentration of 100 μg/mL for each extract, using an R2A medium extract without bacterial inoculation as the control. As shown in **Table** **1**, the MS‐62 extract caused the strongest reduction in PC‐3 cell viability, whereas the MS‐5 extract demonstrated minimal effect.

**Table 1:** Inhibitory effect of 85% methanol extracts from *Micromonospora* strains on the viability of PC‐3 cells at 100 μg/mL.

Strain	Cell viability [%]a)
MS‐3	92.03 ± 3.06
MS‐4	73.01 ± 3.92
MS‐5	87.17 ± 3.92
MS‐11	90.33 ± 2.97
MS‐14	113.64 ± 5.30
MS‐62	44.17 ± 5.67
MS‐209	109.47 ± 4.36
Control(R2A)	75.67 ± 0.94

a) Cell viability represent mean ± SD from three independent experiments (*n* = 3).

Phylogenetic analysis revealed that MS‐62 shared 99.7% 16S rRNA sequence identity with *Micromonospora humi*, although genome‐based comparison suggested that it may represent a new species. Based on its activity, MS‐62 was selected for further investigation as the first *Micromonospora* strain isolated from freshwater sponges to exhibit small molecule mediated inhibition of cancer‐relevant protein targets. MS‐62 (FBCC‐B8445) strain was routinely subcultured on R2A agar at 25 °C and stored at –70 °C in R2A broth supplemented with 40% v/v glycerol.

### 2.2 Identification of Novel Compounds from MS‐62 Strain

A 400‐L culture of the MS‐62 strain was prepared to isolate compounds that reduce cell viability. The 14‐day‐old culture broth was freeze‐dried for 3 days and subjected to sequential extraction with dichloromethane, methanol, and hexane using organic solvents to remove nontarget compounds. Among them, the methanol extract contained compounds that significantly reduced cell viability in the PC‐3 cell line (Table 1). Size‐exclusion chromatography was used to separate the methanol extract into three fractions: M1, M2, and M3. Cell viability assays identified the M2 fraction (260 mg) as the active component, prompting further isolation efforts.

**Figure 3:**
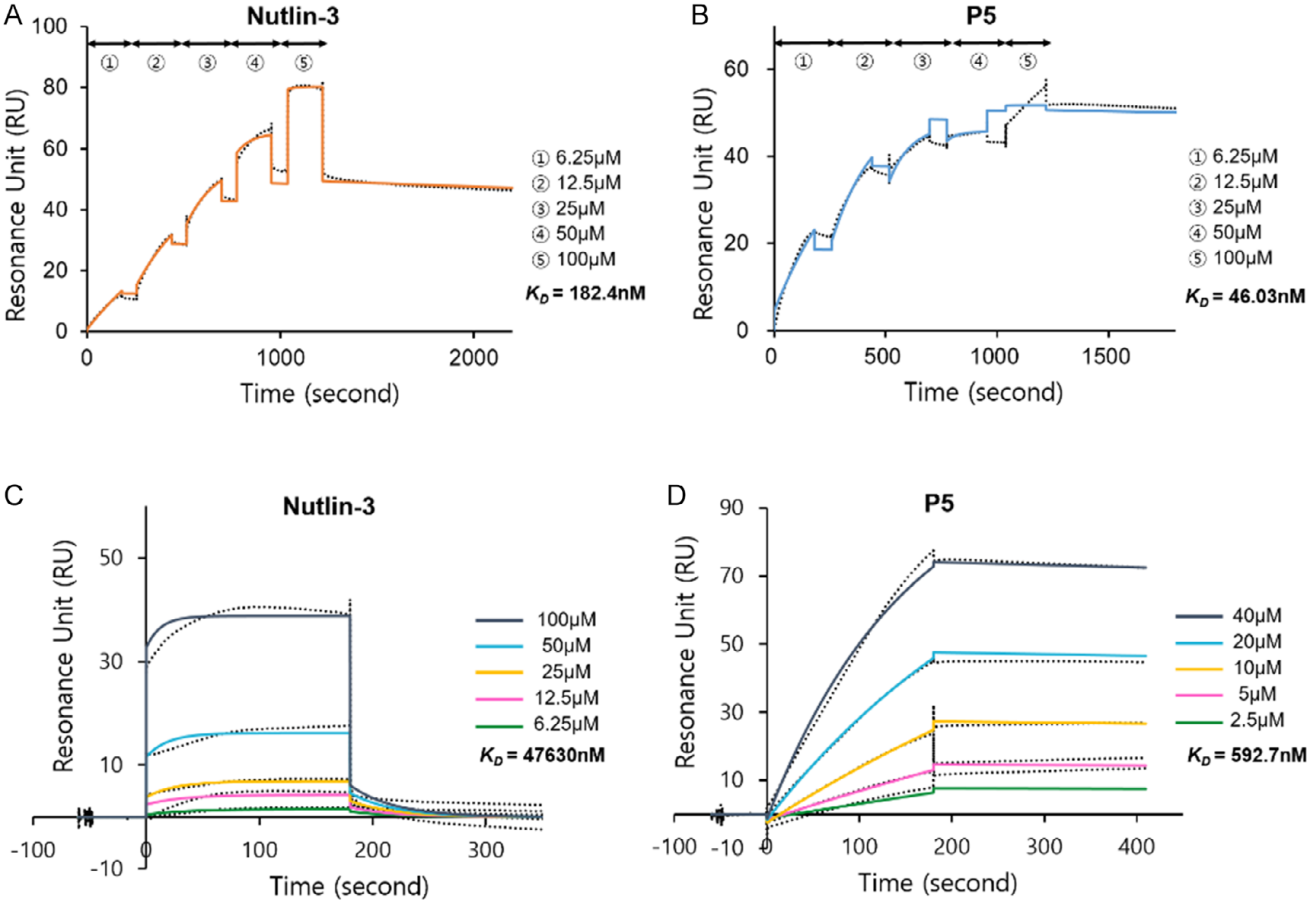
Surface plasmon resonance analysis of A,B) MDM2 and C,D) MDMX binding by (B,D) P5 and the (A,C) reference compound Nutlin‐3, with sensorgram traces represented as dotted lines and fitted curves as solid lines.

High‐performance liquid chromatography with a C8 column revealed nine peaks (Figure 3, Supporting Information). The first three peaks, with a retention time of 18 min, were combined into a single fraction, while the fourth and fifth peaks, at 22 min, were pooled into another fraction. Further peaks at 25, 26, 27, and 31 min were individually fractionated, resulting in six distinct fractions. No reduction in cell viability was detected in the first fraction at 18 min, suggesting that the compounds of interest are associated with the later‐eluting peaks (data not shown). Except for this fraction, the remaining four fractions were purified using isocratic chromatography, yielding five compounds: P1 (0.7 mg), P2 (1.0 mg), P3 (2 mg), P4 (2.5 mg), and P5 (6.5 mg).

**Figure 4:**
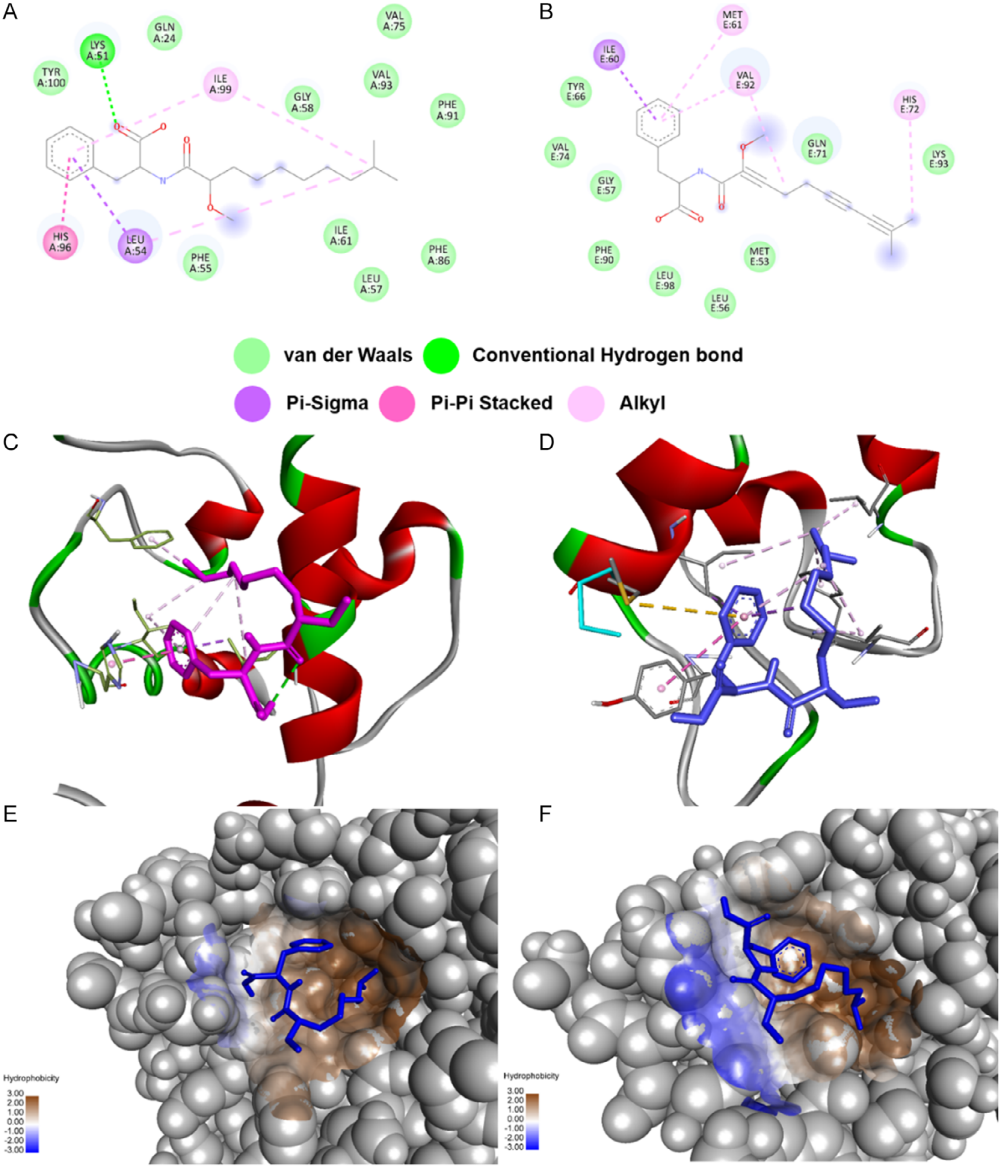
2D and 3D interaction diagrams of compound P5 with A,C,E) MDM2 and B,D,F) MDMX, illustrating hydrogen bonding, π–π stacking, and hydrophobic interactions. (E) and (F) display hydrophobic interaction surfaces.

The ^1^H NMR spectra of these five compounds (Figure 4–7B and 10B, Supporting Information) showed high similarity, commonly characterized by signals at 7 ppm, indicative of a benzene ring, overlapping signals in the shielded region, one methoxy group, and one methyl group. A distinct difference was observed in the methyl proton signals: P1, P3, and P5 exhibited doublet signals, while P2 and P4 showed triplet signals. The detailed structures of these five compounds were established based on the structure determination of compound P5, which was isolated in relatively larger quantities.

The molecular formula of P5 was determined as C_21_H_33_NO_4_ based on its ion peak ([M−H]^−^, *m*/*z* = 362.2322, Δ = −4.1 ppm) in the negative HR‐ESI MS spectrum and the observed carbon signals in the ^13^C spectrum (Figure 8 A, Supporting Information). The ^13^C NMR and HSQC spectra revealed 1 carbonyl carbon (*δ*_C_ 174.8), 4 olefinic carbons (*δ*_C_ 127.6, 129.3, 130.5, and 138.8), 1 oxymethine (*δ*_C_ 83.5), 1 nitrogen bearing carbon (*δ*_C_ 54.9), 10 aliphatic carbons (*δ*_C_ 23.0 × 2, 25.8, 28.5, 29.2, 30.6, 30.8, 33.9, 38.8, and 40.2), and 1 methoxy group (*δ*_C_ 58.4) (Figure 12, Supporting Information). The ^1^H spectrum displayed an intensive methyl signal (*δ*_H_ 0.88), one methoxy signal (*δ*_H_ 3.27), one oxymethine proton (*δ*_H_ 3.51), one aminomethine proton (*δ*_H_ 4.71), benzene ring protons (*δ*_H_ 7.17, 7.21, and 7.24), and several aliphatic chain protons (**Table** **2**). The presence of a benzene ring was confirmed by the proton chemical shifts in the ^1^H spectrum and UV absorbance (254 nm) in the UV spectrum. The COSY spectrum showed a coupling between H‐2 and H‐3, H‐2′ and H‐3′, and H‐9′ and H‐10′ (Figure 11, Supporting Information). The HMBC spectrum revealed correlations between H‐3 and C‐4/C‐5/(9), indicating benzene ring linkage at C‐3 (Figure 13, Supporting Information). The chemical shift of C‐2 suggested a linkage with an amine group at C‐2. Additionally, H‐2′ correlated with the carbonyl carbon (*δ*_C_ 174.8) and the methoxy carbon (*δ*_C_ 58.4) in the HMBC spectrum. Furthermore, the H‐2′ was linked with a long carbon chain terminated by a dimethyl group, as supported by the COSY and TOCSY spectra. Though weak, the HMBC correlation between H‐2 and C‐1′ suggested a linkage with a partial structure of the aliphatic chain. The remaining carboxylic group, inferred from the molecular formula, was connected by the HMBC correlations between H‐3 and an unassigned carbonyl carbon (*δ*_C_ 177.5). Thus, compound P5 was identified as N‐(2‐methyl‐9‐methyldecanoyl)‐phenylalanine (**Figure** **2**). The stereochemistry of phenylalanine was determined as an *L*‐form by comparing the CD spectrum of P5 with the reported spectrum (Figure 9B, Supporting Information).^[^ ^14^ ^]^

**Table 2:** NMR spectral data for compound P5 (solvent: CD_3_OD, 500 MHz for ^1^H).

Number	^13^C	^1^H
1	177.5, C	–
2	54.9, CH_2_	4.71, m
3	38.7, CH_2_	3.01, dd(13.7, 9.1); 3.26, dd(13.7, 4.4)
4	138.8, C	–
5, 9	130.5, CH	7.21, m
6, 8	129.3, CH	7.24, m
7	127.6, CH	7.17, m
1′	174.8, C	–
2′	83.5, CH	3.51, dd(7.1, 5.1)
3′	33.9, CH_2_	1.46, d(3.4)
4′	25.8, CH_2_	1.18, m
5′	30.6, CH_2_	1.29, m
6′	30.8, CH_2_	1.21, m
7′	28.5, CH_2_	1.25, m
8′	40.2, CH_2_	1.17, m
9′	29.2, CH	1.52, m
10′, 11′	23.0, CH_3_	0.88, d(6.6)
2′‐OMe	58.4, CH_3_	3.27, s

Based on the ^1^H and ^13^C NMR spectra, the five compounds shared an identical core skeleton. The difference lay in the length of an aliphatic carbon chain and the nature of the terminal group, which was either linear or branched. These structural variations were determined through HR‐ESI MS analysis in combination with ^1^H and ^13^C NMR spectra. The molecular formulas of compounds, P1 and P2, were determined as C_19_H_29_NO_4_, while P3 and P4 were assigned as C_21_H_31_NO_4_.

### 2.3 Binding Affinity of P5 to MDM2/MDMX

Among the five isolated compounds, P5 was obtained in sufficient quantity and was thus selected for further investigation. Surface plasmon resonance (SPR) analysis was conducted to determine whether P5 directly binds to MDM2 and MDMX and to characterize its binding kinetics under defined conditions.^[^ ^15^ ^]^ Because interactions between p53 and its negative regulators, MDM2 and MDMX, can be influenced by stromal elements and are difficult to faithfully recapitulate in cell‐based systems,^[^ ^6^ ^]^ a label‐free biophysical method such as SPR was employed to directly assess the binding of P5 under defined conditions. MDM2 and MDMX proteins containing the p53‐binding site were immobilized on a CM chip, and the binding profiles of P5 were compared to those of control compounds nutlin‐3. To characterize the binding kinetics of P5, single‐cycle kinetic (SCK) analysis was used for MDM2 due to its instability, while multicycle kinetic (MCK) analysis was applied to MDMX, which was regenerable.^[^ ^16^ ^]^ Quantitative data from sensorgram curves, including the association rate constant (*K*_*a*_), dissociation rate constant (*K*_*d*_), and equilibrium dissociation constant (*K*_*D*_), were obtained by fitting the affinity curves. The *K*_*D*_ values were calculated as the ratio of *K*_*d*_ to *K*_*a*_. The results are summarized in **Figure** **3** and **Table** **3**.

**Table 3:** Binding kinetics of compound P5 to MDM2 and MDMX.

Compounds	MDM2				MDMX				Reference
	*K*_*a*_ [*M* ^−1^ s^−1^]a)	*K*_*d*_ [s^−1^]b)	Rmax [RU]	*K*_*D*_ c)	*K*_*a*_ [*M* ^−1^ s^−1^]	*K*_*d*_ [s^−1^]	Rmax [RU]	*K*_*D*_	
p53	NDd)	ND	ND	1.11 μM	ND	ND	ND	0.34 μM	17
RG7112	ND	ND	ND	10.7 nM	ND	ND	ND	ND	18
CTX1	ND	ND	ND	ND	ND	ND	ND	450 nM	19
RO‐5963	ND	ND	ND	17.3 nM	ND	ND	ND	24.7 nM	20
ALRN‐6924	1.25 × 10^−4^	1.36 × 10^−4^	ND	10.8 nM	5.50 × 10^−4^	3.11 × 10^−3^	ND	57 nM	8
Nutlin‐3	262	4.78 × 10^−5^	49.49	182.4 nM	5.38 × 10^2^	2.56 × 10^−2^	9.01	47 630 nM	This study
P5	408.1	1.88 × 10^−5^	50.63	46.03 nM	1.63 × 10^2^	9.66 × 10^−5^	108	592.7 nM	This study

a) *K*_*a*_ = association rate constant

b) *K*_*d*_ = dissociation rate constant

c) *K*_*D*_ = equilibrium dissociation constant

d) Data not determined

As shown in Figure 3 and Table 3, for MDM2, P5 exhibited a faster association rate (*K*_*a*_: 408.1 M^−^^1^ s^−^^1^ compared to 262 M^−^^1^ s^−^^1^ for Nutlin‐3) and a significantly slower dissociation rate (*K*_*d*_: 1.878 × 10^−^^5^ s^−^^1^ compared to 4.778 × 10^−^^5^ s^−^^1^ for Nutlin‐3), resulting in a much lower equilibrium dissociation constant (*K*_*D*_: 46.03 nM compared to 182.4 nM for Nutlin‐3). For MDMX, P5 exhibited a slower association rate (*K*_*a*_: 1.630 × 10^2^ M^−^^1^ s^−^^1^ compared to 5.378 × 10^2^ M^−^^1^s^−^^1^ for Nutlin‐3) but a substantially slower dissociation rate (*K*_*d*_: 9.661 × 10^−^^5^ s^−^^1^ compared to 2.562 × 10^−^^2^ s^−^^1^ for Nutlin‐3), leading to a significantly lower *K*_*D*_ (592.7 nM compared to 47 630 nM for Nutlin‐3).^[^ ^16^ ^]^ The *K*_*D*_ values obtained for Nutlin‐3 were consistent with previously reported data, validating the suitability of the experimental conditions employed in the SPR analysis, including protein immobilization method and density, injection and dissociation times, flow rate, analyte concentration range, and solvent composition.^[^ ^16^, ^17^ ^]^ SPR analysis indicated that P5 bound both MDM2 and MDMX with higher affinity than Nutlin‐3. P5 also exhibited stronger binding affinity to both MD^1^M2 and MDMX compared with the natural p53 peptide. Specifically, P5 exhibited a significantly higher binding affinity for MDM2 than the natural p53 peptide (1.11 μM) and for MDMX (0.34 μM).^[^ ^18^ ^]^

Compared with other inhibitors, P5 presents distinct advantages and limitations. For instance, CTX1, an MDMX‐specific inhibitor, demonstrated higher inhibitory efficacy than P5 against MDMX (*K*_*D*_: 450 nM)^[^ ^19^ ^]^ and exhibited no detectable binding to MDM2. By comparison, RG7112, a potent MDM2 inhibitor with a *K*_*D*_ of 10.7 nM, exhibited no inhibitory efficacy against MDMX.^[^ ^20^ ^]^ RO‐5963, another dual inhibitor, has been reported to bind more strongly to both MDM2 and MDMX than P5 but suffers from rapid dissociation kinetics, resulting in reduced target engagement duration.^[^ ^8^ ^]^ ALRN‐6924, a stapled peptide currently under clinical evaluation, exhibits dual‐target engagement with high binding affinity and sustained interaction.^[^ ^8^ ^]^ Nevertheless, no small molecule dual inhibitor with comparable characteristics has been reported to date. This comparative analysis underscores P5's key advantage as a compound dual inhibitor capable of targeting both MDM2 and MDMX with favorable binding kinetics, differentiating it from single‐target inhibitors such as CTX1 and RG7112, as well as existing dual inhibitors with pharmacokinetic or kinetic limitations.

### 2.4 Molecular Docking of P5 to MDM2/MDMX

Molecular docking simulations were performed for P5 and Nutlin‐3 to support the interpretation of their experimentally determined binding affinities. AutoDock Vina was used to examine their binding modes within the p53‐binding pockets of MDM2 (PDB ID: 4HG7) and MDMX (PDB ID: 3FE7). These noncatalytic sites mediate protein–protein interactions with p53 and are suitable for small molecule targeting.^[^ ^6^ ^]^ The docking analysis provided structural insights into the orientation and interaction profile of P5 at both targets.^[^ ^21^ ^]^

As summarized in **Table** **4**, P5 exhibited binding energies of –6.9 kcal mol^−1^ with MDM2 and–5.5 kcal mol^−1^ with MDMX, which were lower than those of the well‐characterized MDM2 inhibitors Nutlin‐3 and Nutlin‐3a. Nevertheless, P5 formed a broad network of noncovalent contacts at both targets. In MDM2, it interacted with Leu54, Leu57, Gly58, Ile61, Met62, Tyr67, Gln72, Val75, Phe86, Phe91, Val93, His96, Ile99, Tyr100, and Ile101 through van der Waals forces and hydrogen bonds. π–π stacking between its aromatic ring and His96 enhanced binding stabilization within the hydrophobic pocket. In MDMX, P5 bound to Met53, Leu56, Gly57, Ile60, Met61, Tyr66, Gln71, His72, Val92, Pro95, and Tyr99 via a comparable set of interactions, including π–alkyl and alkyl contacts. Several residues engaged by P5, including Leu54, Leu57, and Tyr100, are also targeted by Nutlin‐3 and Nutlin‐3a. These positions correspond to p53 contact sites (Phe19, Trp23, and Leu26), which are known to contribute to binding affinity by occupying key hydrophobic subpockets in MDM2 and MDMX.^[^ ^7^, ^22^, ^23^ ^]^ **Figure** **4** illustrates the predicted binding poses of P5 within the p53‐binding regions of both proteins.

**Table 4:** Molecular docking analysis of compound P5 with MDM2 (4HG7) and MDMX (3FE7).

Number	Compounds	Binding energy [kcal/mol]	
		MDM2 (4HG7)	Interacting amino acid residues
1	Nutlin‐3	−8.1	Leu54, Phe55, Gly58, Gln59, Ile61, Met62, Tyr67, Val75, Phe91, Val93, His96, Ile99, Tyr100
2	Nutlin‐3a	−7.9	Gln24, Lys51, Leu54, Phe55, Leu57, Gly58, Gln59, Ile61, Gln72, Phe91, Val93, His96, Ile99, Tyr100
3	P5	−6.9	Gln24, Lys51, Leu54, Phe55, Leu57, Gly58, Ile61, Val75, Phe86, Phe91, Val93, His96, Ile99, Tyr100

Number	Compounds	Binding energy [kcal/mol]	
		MDMX (3FE7)	Interacting amino acid residues
1	Nutlin‐3	−6.7	Lys50, Met53, His54, Gly57, Ile60, Met61, Tyr66, Gln71, Val74, Phe90, Val92, Pro95, Leu98
2	Nutlin‐3a	−6.4	Met53, His54, Leu56, Gly57, Ile60, Met61, Tyr66, Gln71, Phe90, Val92, Leu98
3	P5	−5.5	Leu56, Gly57, Ile60, Met61, Tyr66, Gln71, His72, Val74, Phe90, Val92, Pro95, Leu98

Prior structural studies have demonstrated that van der Waals and hydrophobic forces are the dominant contributors to ligand binding at these sites,^[^ ^24^, ^25^ ^]^ and the current docking results are consistent with these findings. Although structurally distinct, P5 occupies comparable positions within the target interface, engaging residues critical for p53 association. Unlike previous strategies that improved MDMX affinity through rigidification or scaffold expansion of Nutlin derivatives,^[^ ^23^ ^]^ P5 achieved dual MDM2/MDMX engagement by forming an extensive interaction network. These findings suggest that P5 represents a structurally distinct scaffold capable of dual engagement with MDM2 and MDMX, offering an alternative to existing chemotypes while maintaining key target interactions. Given the structural similarity of P1–P5, comparable docking results were observed for P1–P4 (Table 1 and Figure 14–16).

However, docking simulations have inherent limitations, such as neglect of molecular flexibility, solvation effects, and the resolution constraints of available crystal structures.^[^ ^24^, ^26^, ^27^ ^]^ Moreover, the limited diversity of MDM2 and MDMX crystal structures available in the PDB may restrict conformational sampling. Nevertheless, the high binding affinity of P5 observed in SPR analysis may be rationalized by the extensive noncovalent interactions predicted in the docking model. This structural rationale supports the plausibility of the proposed binding mode and underscores the complementary role of docking analysis.

### 2.5 Effects of P5 on Cell Viability

**Figure 5:**
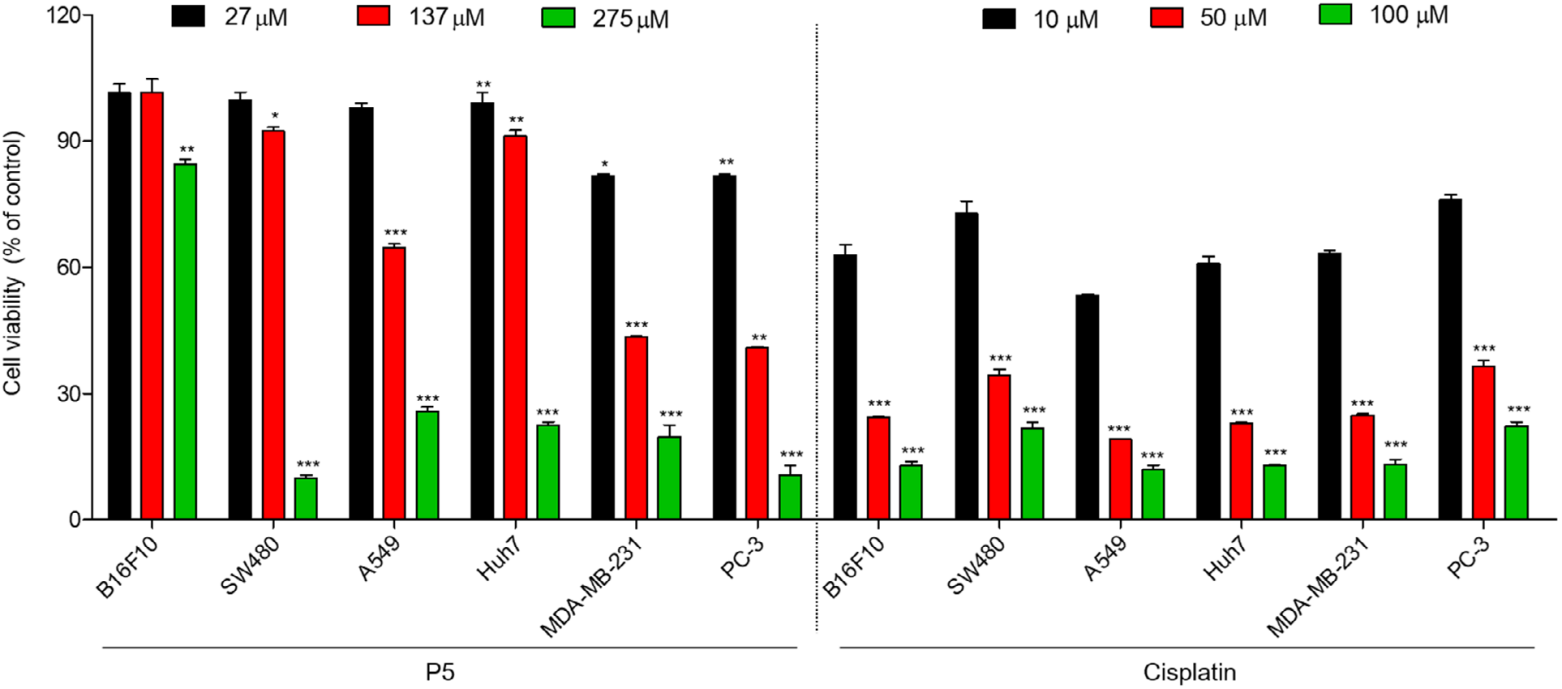
Cell viability of P5 and cisplatin in six cancer cell lines. Cell viability of cancer cells treated with increasing doses of P5 (27, 137, and 275 μM) and cisplatin (10, 50, and 100 μM) for 24 h measured by CCK‐8 assay. Data represent the mean ± SD (*n* = 3), and statistical significance was determined using an unpaired two‐tailed Student's *t*‐test versus the untreated control group (***p < 0.001, **p < 0.01, and *p < 0.05). Cisplatin was used as a positive control.

The newly identified compound P5, which exhibits nanomolar binding affinity to both MDM2 and MDMX, was subjected to an initial biological evaluation using different human cancer cell lines. As shown in **Figure** **5**, P5 reduced cell viability in a concentration‐dependent manner across all tested cell lines. Based on IC_50_ values, PC‐3 and MDA‐MB‐231 cells were the most sensitive to P5 treatment, with IC_50_ values of 30.24 ± 3.85 μM and 49.33 ± 3.30 μM, respectively. Moderate sensitivity was observed in Huh7 cells (71.21 ± 5.23 μM), while SW480 and A549 cells exhibited lower sensitivity, with IC_50_ values of 72.23 ± 5.78 μM and 73.22 ± 4.13 μM, respectively. Notably, B16F10 melanoma cells showed the lowest sensitivity, with an IC_50_ value exceeding 275 μM. For comparison, the positive control cisplatin exhibited significantly stronger cytotoxicity, with IC_50_ values of 16.21 ± 0.23 (B16F10), 25.2 ± 0.41 (SW480), 12.00 ± 0.22 (A549), 15.22 ± 0.12 (Huh7), 17.71 ± 0.23 (MDA‐MB‐231), and 31.93 ± 0.31 μM (PC‐3), respectively. These results indicate that, while P5 displays notable anticancer activity in several lines, its potency is generally lower than that of cisplatin.

P5 exhibited response patterns similar to those reported for established MDM2/MDMX inhibitors. For instance, the MDM2‐selective inhibitors Nutlin‐3, Idasanutlin (RG7388), MI‐219, and RG7112 have been reported to be effective against p53 wild‐type cell lines, with reduced cellular sensitivity observed against cell lines lacking functional p53, including p53‐null and p53‐mutant cell lines.^[^ ^4^, ^20^, ^23^, ^28^ ^]^ Especially, MI‐219 demonstrated limited efficacy against p53‐mutant cell lines, consistent with the attenuated response of SW480 and the moderate response seen in p53‐null PC‐3 cells.^[^ ^28^ ^]^ Similarly, the MDMX‐selective inhibitor CTX1 exhibited reduced activity in p53‐null cell lines such as HCT116 p53^−^/^−^ and A549 shp53, while maintaining higher efficacy in p53 wild‐type OCI and HCT116 cells.^[^ ^19^ ^]^ In addition, the dual MDM2/MDMX inhibitor RO‐5963 induced apoptotic responses in p53 wild‐type cancer cell lines such as U2OS, AGS, and G401, whereas p53‐mutant or p53‐null cell lines, including SW480, MDA‐MB‐435, and LS174T, were not sensitive to treatment.^[^ ^29^ ^]^ A comparable pattern was observed with the dual MDM2/MDMX inhibitor ALRN‐6924. In p53 wild‐type leukemia cell lines such as BV‐173 and NALM‐6, ALRN‐6924 reduced cell viability, while p53‐mutant cell lines such as Thp1 and HEL 92‐1‐7 exhibited minimal responses.^[^ ^8^ ^]^ These comparative findings suggest that p53 status can influence cellular responses to MDM2/MDMX inhibition. Furthermore, previous studies have demonstrated that MDM2 and MDMX also modulate the p53 homolog p73 under p53‐deficient conditions.^[^ ^30^ ^]^ Given the regulatory complexity involving both p53 and its homologs, further investigation is necessary to elucidate the full spectrum of molecular interactions affected by P5.

### 2.6 In Silico Pharmacokinetic Profile of P5

Several previously developed MDM2/MDMX inhibitors exhibit unfavorable pharmacokinetic profiles, including low gastrointestinal absorption, poor solubility, and limited bioavailability.^[^ ^7^ ^]^ The in silico pharmacokinetic properties of P5 and reference compounds were predicted using SwissADME, a widely used web‐based platform for evaluating physicochemical descriptors and drug‐likeness parameters.^[^ ^31^, ^32^, ^33^ ^]^

The predicted pharmacokinetic parameters are summarized in **Table** **5**. A radar plot highlighting the distribution of key ADME descriptors is provided in Figure 17, Supporting Information. P5 was predicted to exhibit high gastrointestinal (GI) absorption and blood–brain barrier (BBB) permeability, with a bioavailability score of 0.56. It satisfied Lipinski's rule of five as well as Ghose, Egan, and Muegge criteria and triggered no PAINS or Brenk structural alerts. The topological polar surface area (TPSA) of P5 was calculated as 75.63 Å^2^, suggesting favorable passive permeability. P5 was also predicted to be moderately soluble (log *S* = –4.14), whereas Nutlin‐3, Nutlin‐3a, and RG‐7112 were classified as poorly soluble. While P5 violated the Veber rule due to a high number of rotatable bonds, its overall profile suggests good drug‐like potential.

**Table 5:** Prediction of ADME properties of P5 using SwissADME.

Properties		P5 (This study)	Nutlin‐3	Nutlin‐3a	RG‐7112	RO‐5963	CTX1
Physicochemical properties	TPSA (Å^2^)	75.63	83.47	83.47	90.90	139.94	88.72
Water solubility	Log *S* (ESOL)	−4.14	−6.53	−6.53	−8.41	−3.74	−2.84
	Solubility class	Moderately soluble	Poorly soluble	Poorly soluble	Poorly soluble	Soluble	Soluble
Pharmacokinetics	GI absorption	High	High	High	Low	Low	High
	BBB permeant	Yes	No	No	No	No	No
	P‐gp substrate	Yes	Yes	Yes	Yes	Yes	Yes
	CYP1A2 inhibitor	No	No	No	No	No	Yes
	CYP2C19 inhibitor	No	Yes	Yes	No	No	No
	CYP2C9 inhibitor	Yes	Yes	Yes	No	No	No
	CYP2D6 inhibitor	Yes	Yes	Yes	Yes	No	Yes
	CYP3A4 inhibitor	Yes	Yes	Yes	Yes	No	Yes
	Log Kp (skin permeation)	−5.36 cm/s	−6.16 cm/s	−6.16 cm/s	−5.58 cm/s	−8.50 cm/s	−6.63 cm/s
Druglikeness	Lipinski	Yes	No; 2 violations: MW > 500, MLOGP > 4.15	No; 2 violations: MW > 500, MLOGP > 4.15	No; 2 violations: MW > 500, MLOGP > 4.15	Yes; 1 violation: MW > 500	Yes
	Ghose	Yes	No; 2 violations: MW > 480, MR > 130	No; 2 violations: MW > 480, MR > 130	No; 4 violations: MW > 480, WLOGP > 5.6, MR > 130, #atoms > 70	No; 2 violations: MW > 480, MR > 130	Yes
	Veber	No; 1 violation: Rotors > 10	Yes	Yes	No; 1 violation: Rotors > 10	Yes	Yes
	Egan	Yes	Yes	Yes	No; 1 violation: WLOGP > 5.88	No; 1 violation: TPSA > 131.6	Yes
	Muegge	Yes	No; 1 violation: XLOGP3 > 5	No; 1 violation: XLOGP3 > 5	No; 2 violations: MW > 600, XLOGP3 > 5	Yes	Yes
	Bioavailability Score	0.56	0.17	0.17	0.17	0.55	0.55
Medicinal chemistry	PAINS	0 alert	0 alert	0 alert	0 alert	0 alert	0 alert
	Brenk	0 alert	0 alert	0 alert	0 alert	0 alert	2 alert
	Leadlikeness	No; 3 violations: MW > 350, Rotors > 7, XLOGP3 > 3.5	No; 3 violations: MW > 350, Rotors > 7, XLOGP3 > 3.5	No; 3 violations: MW > 350, Rotors > 7, XLOGP3 > 3.5	No; 3 violations: MW > 350, Rotors > 7, XLOGP3 > 3.5	No; 2 violations: MW > 350, Rotors > 7	No; 1 violation: MW < 250
	Synthetic accessibility	3.54	5.16	5.16	6.32	4.60	1.85

ALRN‐6924, a dual MDM2/MDMX inhibitor, was not included in the in silico prediction due to its peptide‐based structure; however, previous reports indicate that it was optimized for solubility and cellular uptake.^[^ ^22^ ^]^ Compared to several existing inhibitors with known ADME liabilities, such as Nutlin‐3, Nutlin‐3a, RG‐7112, RO‐5963, and CTX1, P5 exhibited a more favorable drug‐like profile across multiple parameters. P5 presents a more balanced drug‐like profile, supporting its potential for further development.

## 3 Conclusion

P5, a structurally unique compound isolated from a freshwater sponge‐associated *Micromonospora* sp. MS‐62, was identified as a dual inhibitor of MDM2 and MDMX. It exhibited nanomolar binding to the p53‐binding domains of both targets, as demonstrated by molecular docking and SPR analysis. Additionally, P5 exhibits favorable pharmacokinetic properties and sustained binding kinetics, which distinguish it from previously reported inhibitors. Given the current lack of potent dual MDM2/MDMX inhibitors, P5 represents a promising scaffold for the development of future anticancer therapeutics. Further structural optimization of P5 could enhance its potency and selectivity as a dual‐targeting agent.

## 4 Experimental Section

4.1

4.1.1

##### Material and Methods: Bacterial Strain Isolation from Freshwater Sponge

A freshwater sponge collected from the Geumgang River in Muju, Jeollabuk‐do, Republic of Korea (36°01′03.0″N 127°38′32.6″E) on July 9, 2019. Species identification was conducted based on spicule morphology and CO1 barcode gene sequence analysis. The obtained CO1 sequence was subjected to NCBI BLAST search, and the sponge was identified as *Spongilla lacustris* (supporting information). To prepare the sponge extract, the sample was homogenized using a sterile mortar and pestle with 5 mL of sterile water. The sponge extract was then diluted with 0.8% NaCl (99.5%, Samchun, Pyeongtaek, Korea) in H_2_O and spread onto R2A agar (BD Difco, Sparks, MD, USA) plates. After static incubation for 7 days, pure individual colonies were isolated. A total of 200 colonies were analyzed using 16S rRNA sequencing at Macrogen (Seoul, Korea), resulting in the identification of seven Micromonospora strains. These strains were subsequently inoculated into 10 mL of R2A liquid medium (BD, USA) and cultured at 25 °C for 14 days with agitation at 120 rpm. After cultivation, the culture broth was freeze‐dried and sequentially extracted using dichloromethane (≥99.8%, Sigma–Aldrich, St. Louis, MO, USA), 85% methanol (≥99.8%, Merck, Rahway, NJ, USA), and hexane. The 85% methanol layer was collected and dissolved in DMSO (Molecular biology grade, Sigma–Aldrich, USA) to prepare stock solutions. To assess cell viability, the stock solutions were applied to PC‐3 cells at final concentrations of 50, 100, 500, and 1000 μg/mL. Among the tested strains, the MS‐62 strain demonstrated the strongest reduction in cell viability and was selected for further analysis. Whole‐genome sequencing was performed for MS‐62 for precise species identification. The strain MS‐62 has been deposited with the Freshwater Bioresources Culture Collection (FBCC‐B8445).

##### Purification and Identification of Novel Compounds

The MS‐62 strain was inoculated into a 400‐L fermenter (1.5 ton, KoBioTech Co., Ltd, Seoul, Korea) containing R2A medium and cultured at 25 ± 0.5 °C for 14 days with agitation at 120 rpm, without pH adjustment. Filtered air was supplied at 0.5 v/vm, and the vessel pressure was maintained at 0.3 ± 0.02 bar. Thirty milliliters of sterilized antifoaming agent (LS‐300, Dow Corning, Midland, MI, USA) was added to prevent excessive foam formation. The freeze‐dried culture broth (200 g) was dissolved in 1 L of water and extracted twice over 2 days with an equal volume of dichloromethane (Sigma–Aldrich, USA). The organic dichloromethane layer was subsequently partitioned using 85% methanol (≥99.8%, Merck, USA) and hexane (≥98%, Merck, USA). The hexane layer was discarded, while the 85% methanol layer was collected as it was identified to contain compounds that reduced cell viability. The 85% methanol layer was loaded onto a Sephadex LH‐20 (Cytiva, Marlborough, MA, USA) column and fractionated into three fractions (M1, M2, and M3) using methanol (HPLC grade, Merck, USA) as the eluent. Fraction M2 exhibited reduced cell viability was further purified using high‐performance liquid chromatography (Agilent 1200, Santa Clara, CA, USA) with a Phenomenex C8 column (250 × 10 mm, 5 μm, Torrance, CA, USA) at a flow rate of 2 mL/min. Detection was performed using a refractive index detector, with the mobile phase comprising 20% water and 80% methanol (HPLC grade, Merck, USA), supplemented with 2 mM ammonium acetate (≥97%, Sigma–Aldrich, USA), and 50 mM formic acid (≥96%, Sigma–Aldrich, USA). Five peaks (P1, P2, P3, P4, and P5) were identified and isolated based on the HPLC chromatogram.

The structures of these five compounds were analyzed using ^1^H NMR spectroscopy and high‐resolution electrospray ionization mass spectrometry (X500R, SCIEX, Redwood City, CA, USA). Their molecular structures were elucidated through 2D NMR spectroscopy. Additionally, CD spectroscopy (J‐1500, JASCO, Hachioji, Tokyo, Japan) was employed to confirm the configuration of amino acid residues within the compounds.

**Compound P5**: ^1^H (500 MHz, CD_3_OD) and ^13^C (125 MHz, CD_3_OD) NMR: in Table 2; IR (film) : $\tilde{\nu }$ = 1648 (s), 1523 (s) cm^−1^ (C=O); UV/VIS (acetonitrile) : $\lambda_{\text{max}}$ (ε=217) (8000 mol^−1^ dm^3^cm^−1^; HRMS (ESI) : *m/z* calcd for C_21_H_33_NO_4_: 362.2337 [M−H]^−^; found: 362.2322.

##### Molecular Docking Simulation

The protein structures of MDM2 and MDMX were retrieved from the RCSB Protein Data Bank (https://www.rcsb.org/). Protein structures with resolutions of 2.5 Å or higher were utilized in this study. Specifically, 4HG7 and 3FE7 were selected for their clear and accurate depiction of the active sites and inhibitor interactions. Using AutoDockTools (version 1.5.7),^[^ ^34^ ^]^ co‐crystallized inhibitors were removed from the protein structures, and polar hydrogen atoms and Kollman charges were added to prepare the proteins for docking. The processed protein structures were saved in PDBQT format. The 3D structures of compound P5 were generated using SMILES data output from OpenBabel.^[^ ^35^ ^]^ The structures were further processed and optimized using Avogadro software (version 1.2.0 n). Ligands were energy‐minimized to achieve stable conformations, and PDBQT files were prepared using AutoDockTools. Reference ligand (nutlin‐3 and nutlin‐3a) structures were obtained from PubChem (https://pubchem.ncbi.nlm.nih.gov/) and optimized using Avogadro software to ensure accurate docking simulations.

Docking simulations were performed using AutoDock Vina (version 1.1.2)^[^ ^36^ ^]^ to calculate the binding affinities. The grid box for docking was defined based on the region where the ligand was co‐crystallized. For MDM2, the grid size was set to (*x* = 20 Å, *y* = 20 Å, and *z* = 20 Å) with the center at coordinates (−24.626, 8.167, and −10.472), spacing of 1 Å, and an exhaustiveness parameter of 32. For MDMX, the grid size was set to (*x* = 20 Å, *y* = 24 Å, *z* = 24 Å) with the center at coordinates (22.250, 19.861, and 5.833), spacing of 1 Å, and an exhaustiveness parameter of 32. Only conformations with a root‐mean‐square deviation (RMSD) of 0 were considered for further analysis. Docking results were visualized using AutoDockTools, and the binding interactions were analyzed using BIOVIA Discovery Studio 2024 client software.

##### Binding Kinetics of P5 to MDM2 and MDMX

SPR analysis was conducted using a Biacore T200 system (Cytiva, Uppsala, Sweden) at WOOJUNG BIO, Inc. (Suwon, Korea) to evaluate the binding interactions of compound P5 with MDM2 and MDMX. The p53‐binding domains of MDM2 (ab167941, Abcam, Cambridge, UK) and MDMX (ab167947, Abcam, UK) were preconditioned to 10 mM sodium acetate buffer (pH 5.5, Sigma–Aldrich, USA) and immobilized onto a CM5 sensor chip (Cytiva, Sweden), achieving over 825 RU of immobilized protein. Compound P5 was diluted in running buffer containing 10 mM PBS (pH 7.4, Sigma–Aldrich, USA), 138 mM NaCl (≥99%, Sigma–Aldrich, USA), and 2% DMSO (≥99.5%, Sigma–Aldrich, USA) to final concentrations of 0, 100, and 200 μM and injected at a flow rate of 30 μL min^−1^. Nutlin‐3 (≥98%, Sigma–Aldrich, USA) served as a reference compound for comparative analysis. Binding affinity, represented by the equilibrium dissociation constant (*K*_*D*_), was determined for the immobilized proteins. Further analysis of compound P5 was performed using single‐cycle kinetics with varying concentrations (6.25, 12.5, 25, 50, and 100 μM) under the same flow rate for a duration of 1 min. For multicycle kinetics, varying concentrations of 2.5, 5, 10, 20, and 40 μM were injected for compound P5, while Nutlin‐3 was analyzed at concentrations of 3.125, 6.25, 12.5, 25, 50, and 100 μM. Regeneration of the chip surface was achieved using a solution containing 0.05% Tween 20 (Molecular Biology Grade, Millipore, Bedford, MA, USA), 5, 25, and 50 mM NaOH (≥98%, Sigma–Aldrich, USA).

The SPR signals were analyzed using Biacore T200 Control Software (version 3.2, Cytiva, Sweden) and Biacore T200 Evaluation Software (version 3.2, Cytiva, Sweden) through multicycle kinetic and single‐cycle kinetic models. The *K*_*D*_ values were calculated based on the association rate constant (*K*_*a*_) and dissociation rate constant (*K*_*d*_) using the following equation, and the data were fitted to a 1:1 binding model for calibration.

$$K_{D}\left(M\right)=\;\frac{K_{d}\;\left(s^{-1}\right)}{K_{a}\;\left(M^{-1}\;s^{-1}\right)}$$

##### Biological Evaluation

The B16F10 (mouse melanoma), SW480 (human colon adenocarcinoma), A549 (human lung carcinoma), Huh7 (human hepatocellular carcinoma), MDA‐MB‐231 (human breast adenocarcinoma), and PC‐3 (human prostate carcinoma) cell lines were obtained from the American Type Culture Collection (ATCC, Rockville, MD, USA). The cell lines were cultured in RPMI 1640 medium (Gibco, Gaithersburg, MD, USA) supplemented with 10% fetal bovine serum and 1% penicillin–streptomycin at 37 °C in a humidified incubator with 5% CO_2_. For treatment, the freeze‐dried culture extracts of the strains were dissolved in dimethyl sulfoxide (DMSO; molecular biology grade, Sigma–Aldrich, St. Louis, MO, USA) to prepare a 1 mg mL^−1^ stock solution, which was further diluted to final concentrations of 100, 500, and 1000 μg mL^−1^. The purified compound P5 was dissolved in DMSO to a final stock concentration of 10 mM and applied at 27, 137, and 275 μM. Cisplatin (Sigma–Aldrich, USA) was prepared as a 10 mM DMSO stock solution and used as a positive control at the same concentrations (10, 50, and 100 μM). Cells were seeded at 1 × 10^4^ cells per well in 6‐well plates and treated with either the extracts, P5, and cisplatin for 24 h. For cell viability assessment, CCK‐8 reagent (Sigma–Aldrich, USA) was added to each well at a final concentration of 0.5 mg/mL and incubated for 1 h at 37 °C. The medium was then removed, and 2 mL of DMSO was added to solubilize the formazan crystals. Absorbance was measured at 540 nm using a microplate reader (Molecular Devices, Sunnyvale, CA, USA).

##### Statistical Analysis

All experiments were performed in triplicate unless otherwise specified. Quantitative data are presented as mean ± standard deviation. Statistical comparisons between treated and untreated groups were conducted using an unpaired two‐tailed Student's *t*‐test. Statistical significance was defined as *p* < 0.05 (*), *p* < 0.01 (**), and *p* < 0.001 (***). All analyses were performed using GraphPad Prism (version 10.4.2, San Diego, CA, USA).

## Conflict of Interest

The authors declare no conflict of interest.

## Supporting information

Supplementary Material

## Data Availability

The data that support the findings of this study are available from the corresponding author upon reasonable request.
