# Supplementary material for: Discovery of a Phenylalanine‐Derived Natural Compound as a Potential Dual Inhibitor of MDM2 and MDMX
Source: ChemMedChem. 2025 Jul 22;20(16):e202500397. doi: 10.1002/cmdc.202500397 (PMC12368481; doi:10.1002/cmdc.202500397)
Supplement: Supplementary file 1 — Supplementary Material [file CMDC-20-e202500397-s001.pdf]

Supporting Information

Co1 sequencing of collected freshwater sponge

GGTGACCAAAGAATCAAAATAAATGTTGAAATAGTATTGGGTCTCCTCCTCCAGCAGGATCAAAG
AATGTTGTATTAAAATTTCTATCTGTTAAAAGCATTGTTATACCACCAGCTAATACAGGTAAAGATA
ATAATAATAAAAAGGCTGTTATTAAAATAGATCATACAAATAATGGCATTCTATCCATTGTAATACC
GGGCGCTCTCATATTAAAGATTGTTGTGATAAAATTCATAGCCCCTAATATCGAAGAAATACCCGC
CAAGTGAAGACTAAATATTGCCATATCAACTGATCCCCCAGAATGTGCTTGTATGCCTGCTAAAG
GGGGATATACTGTCCATCCGGTACCAACCCCTTGCTCTACAAAAGCAGATCCTAATAATAGAGTT
AAAGCCGGAGGTAATAATCAAAAACTAATATTGTTTAATCTTGGAAAAGCCATATCGGGTGCACC
AATATATAATGGCACGAATCAATTTCCAAATCCCCCAATCATTACTGGCATAACTAAGAAAAATAT
CATTAGAAAAGCATGGGCTGTAACTATAACATTATATAATTGATCATCCCCTAACATTGACCCAGG
GGCTGATAGCTCTAATCTTATAAGCATACTAAATGCTGTACCTATCATGCCTGCAAATGCTCCAAA
TAATAAATAAAGAGTCCCGATATCTTTATGTTTTGTTGAACCA

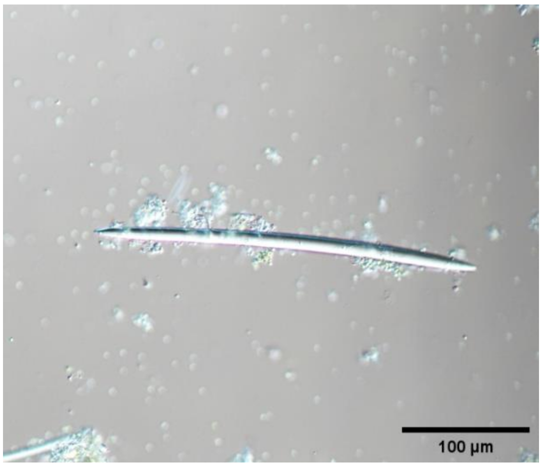

Supplementary Figure 1. Spicule of collected freshwater sponge. Scale bar represents 100 µm

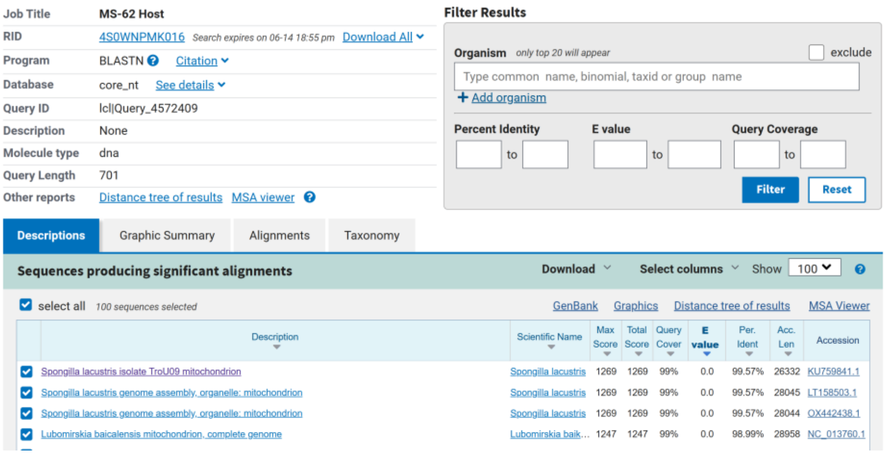

Supplementary Figure 2. NCBI Blast result of obtained Co1 sequence

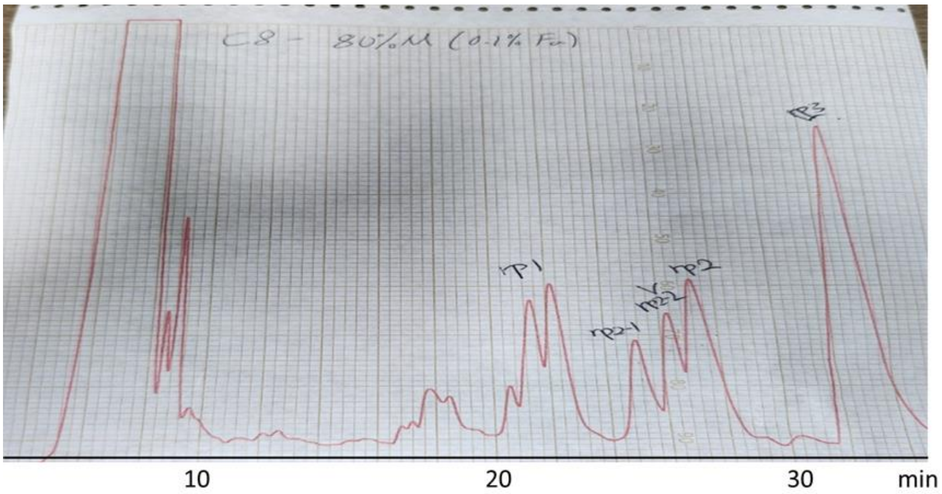

Supplementary Figure 3. Chromatogram of fraction M2

A

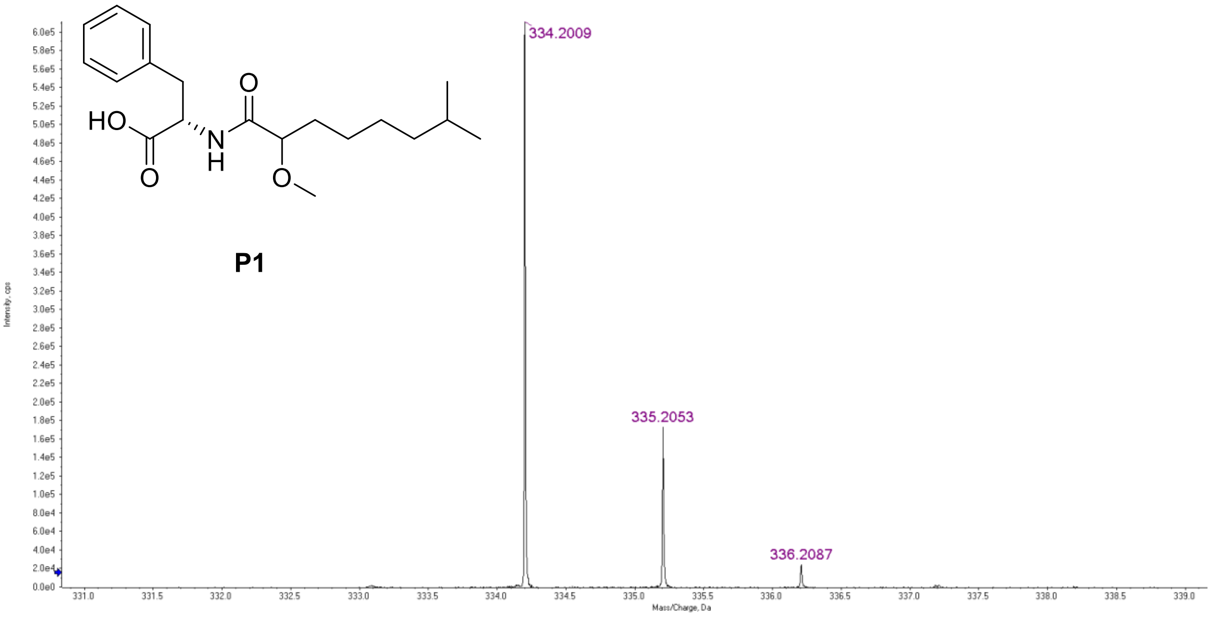

B

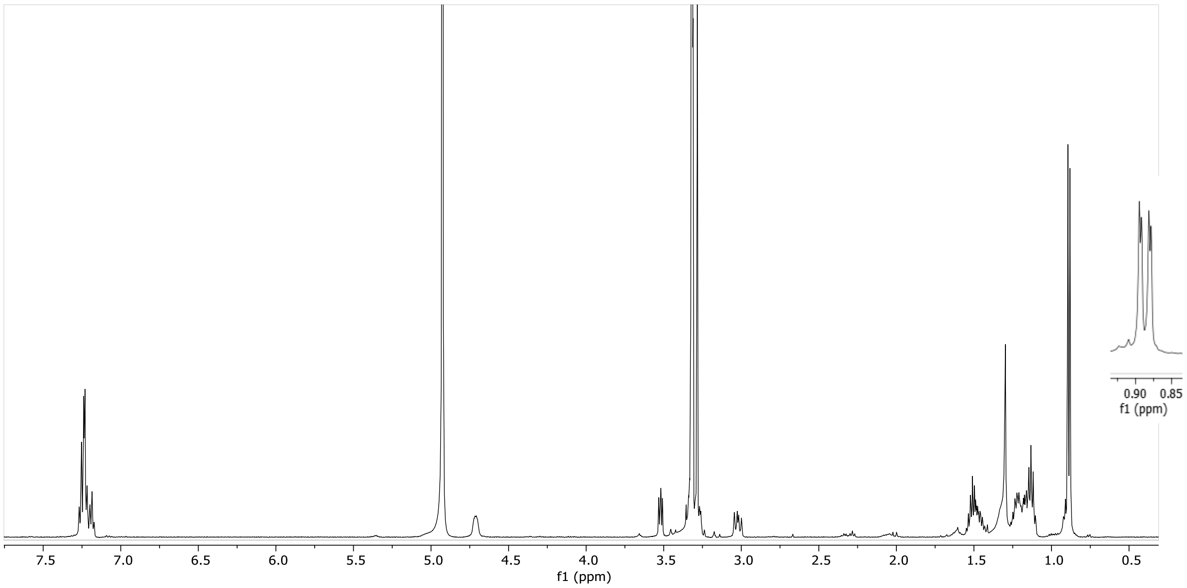

Supplementary Figure 4. (A) HRMS data and (B) ^{1}H NMR spectrum of P1 at 500 MHz

A

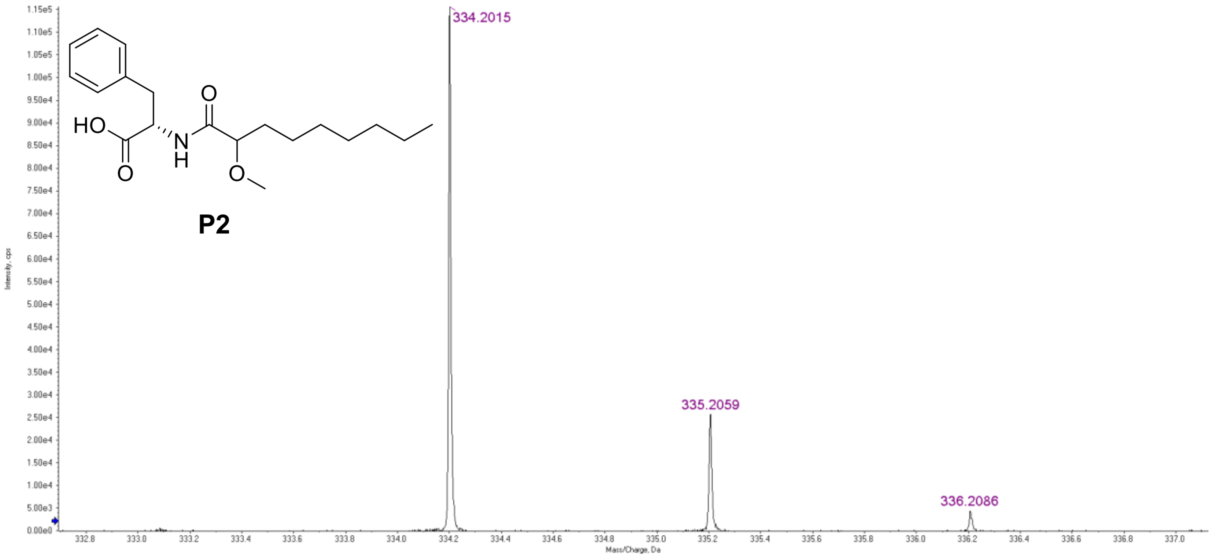

B

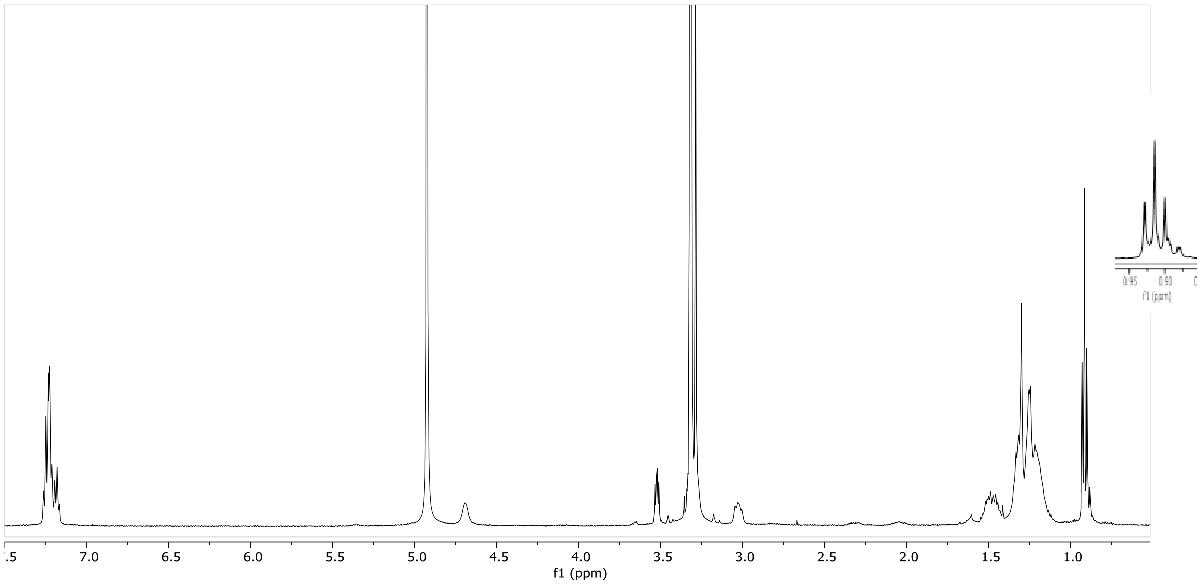

Supplementary Figure 5. (A) HRMS data and (B) ^{1}H NMR spectrum of P2 at 500 MHz

A

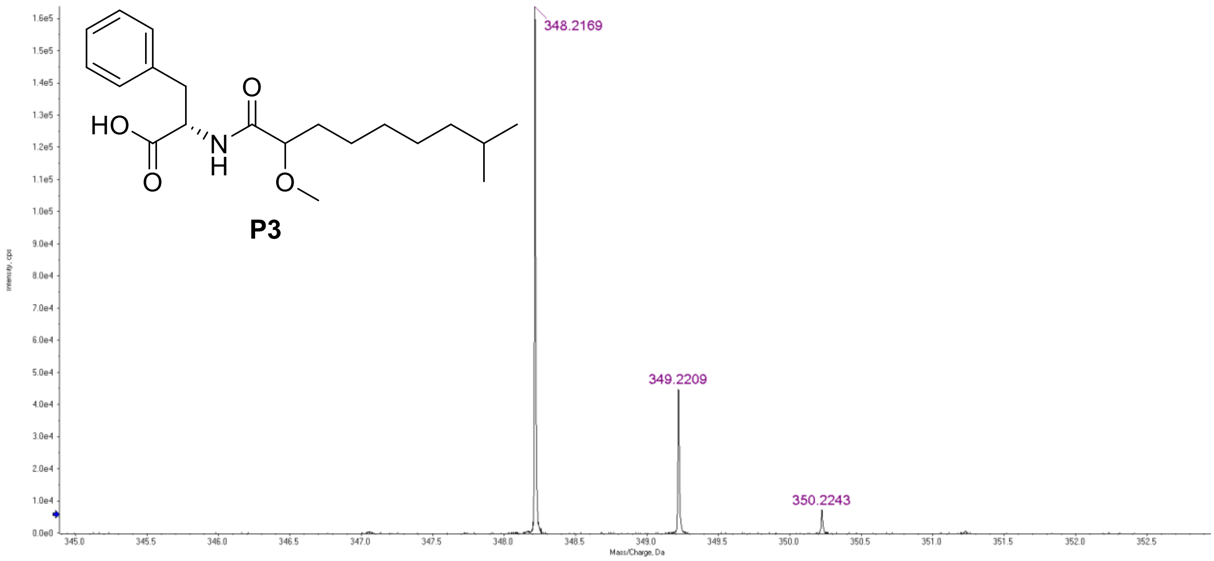

B

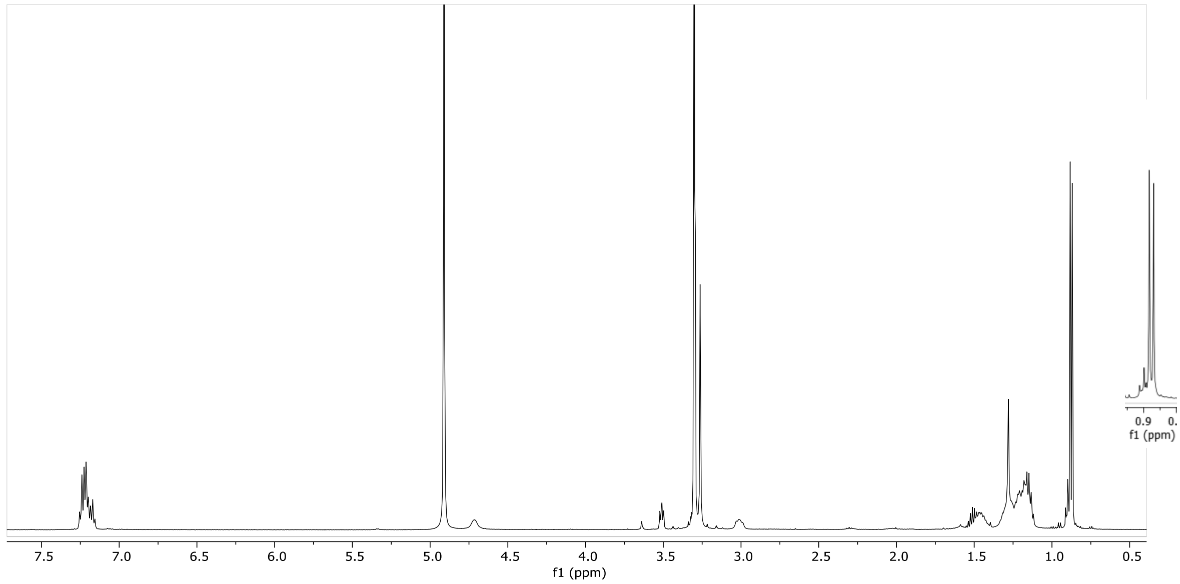

Supplementary Figure 6. (A) HRMS data and (B) ^{1}H NMR spectrum of P3 at 500 MHz

A

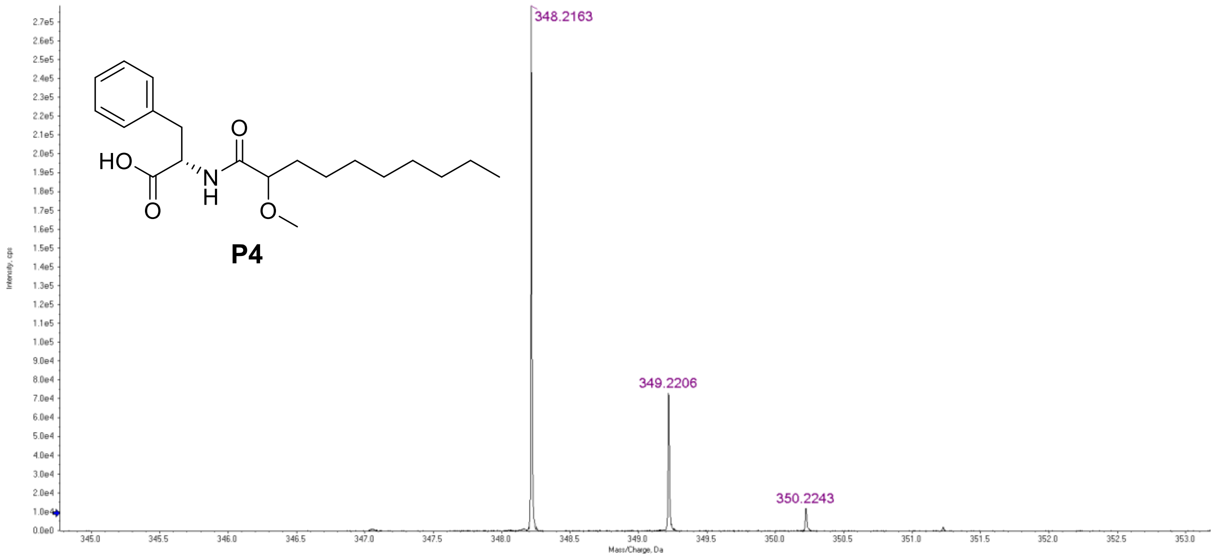

B

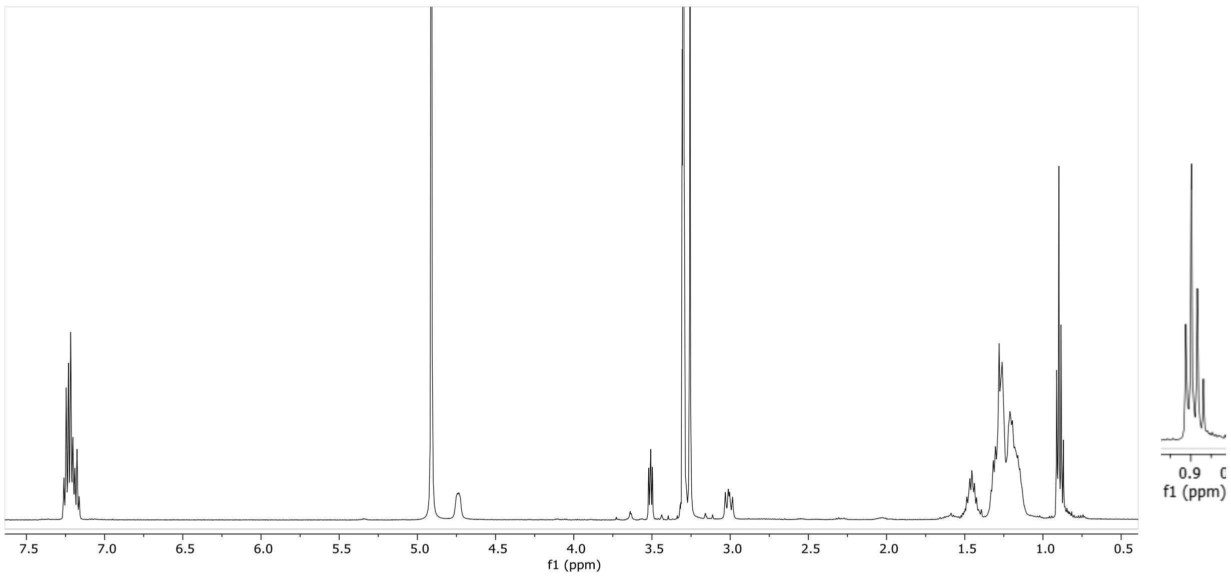

Supplementary Figure 7. (A) HRMS data and (B) ^{1}H NMR spectrum of P4 at 500 MHz

A

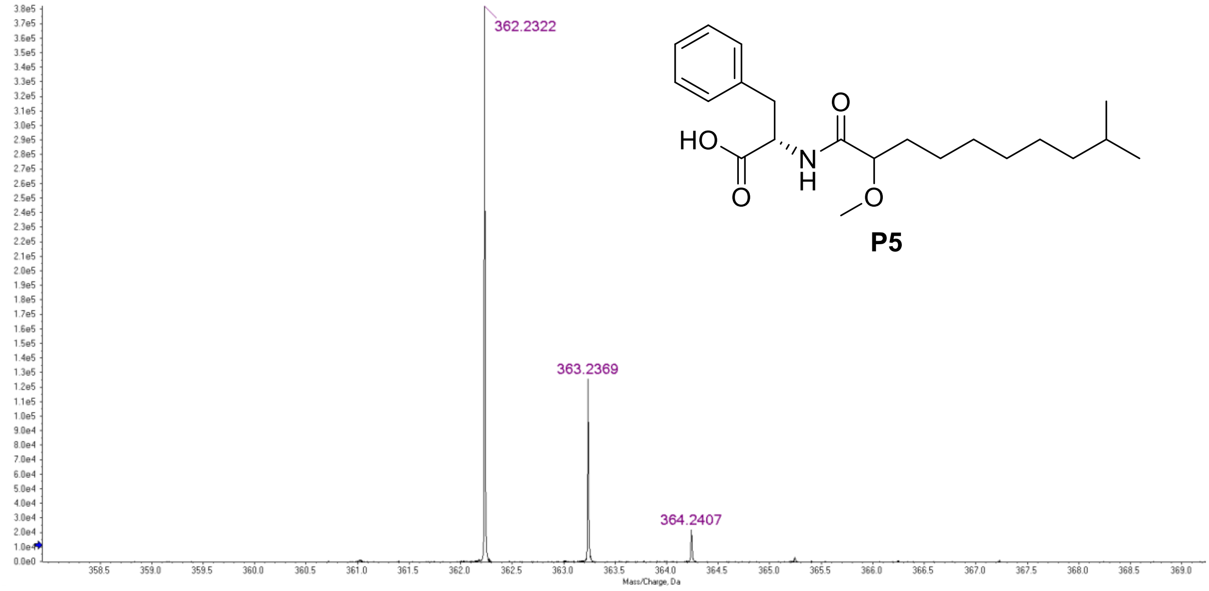

B

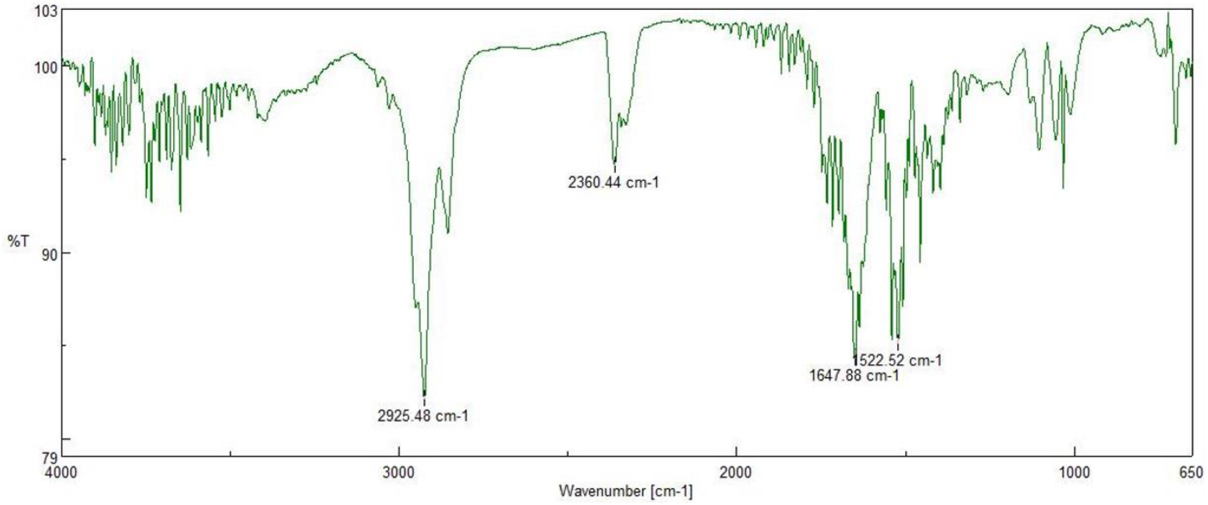

Supplementary Figure 8. (A) HRMS data and (B) IR spectrum of P5

A

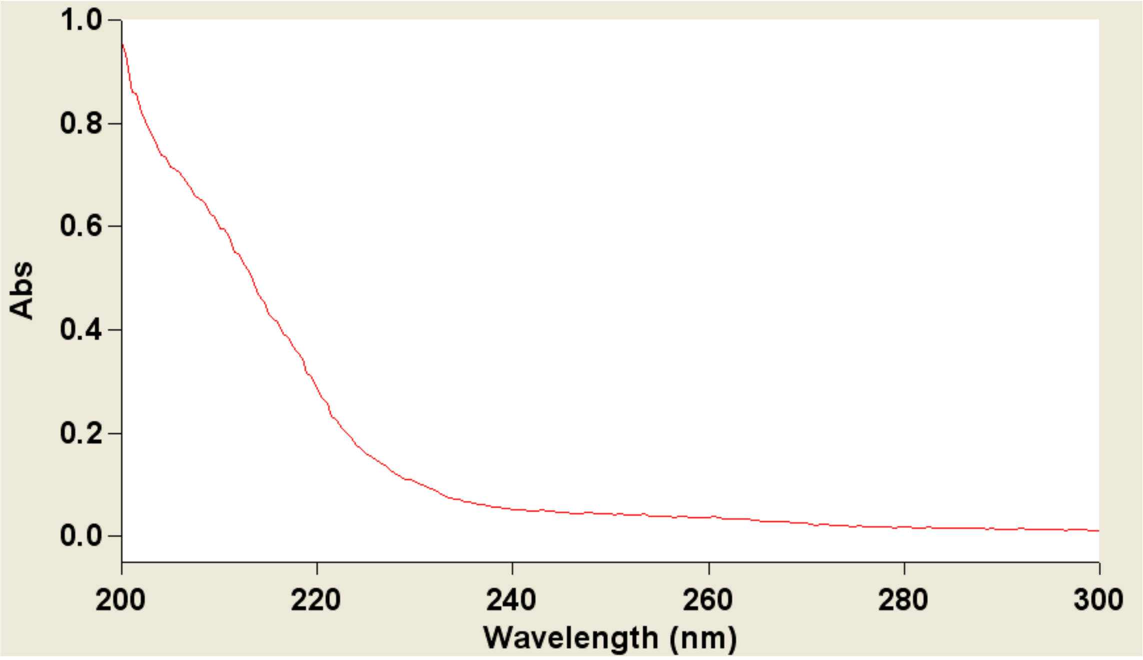

B

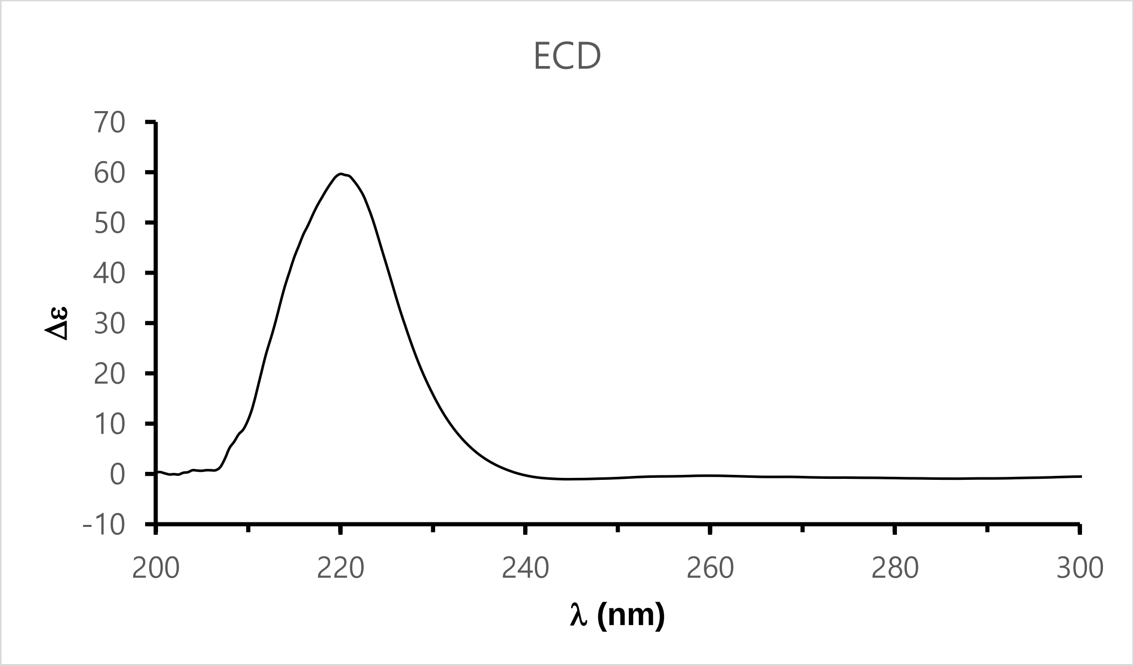

Supplementary Figure 9. (A) UV/VIS and (B) ECD spectra of P5

A

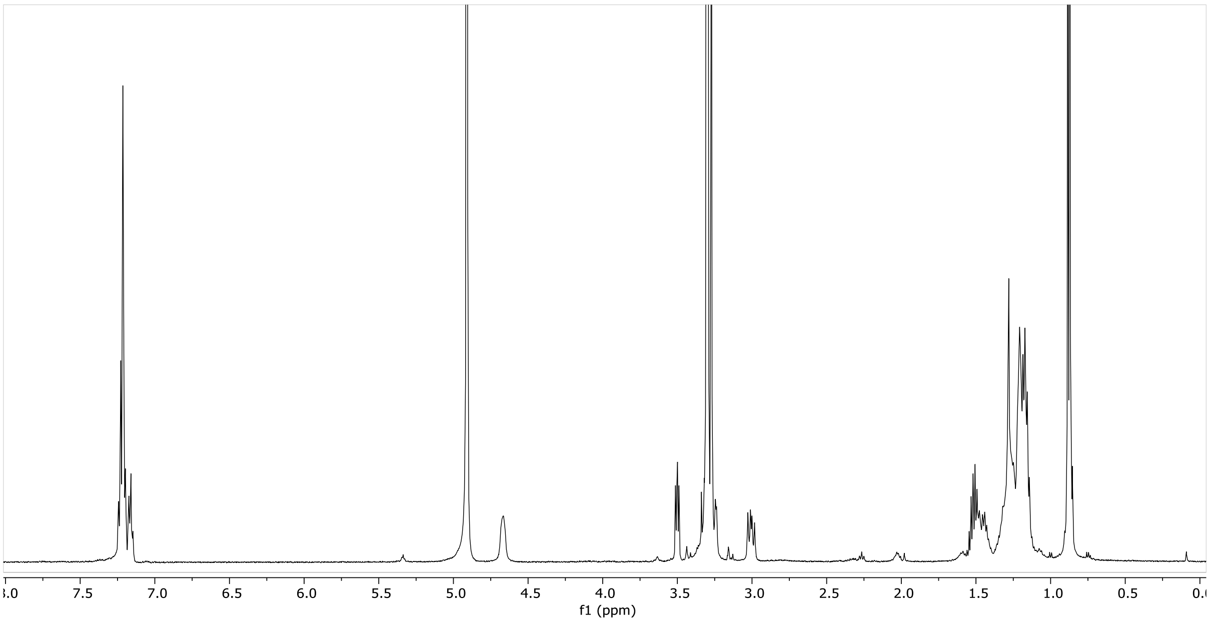

B

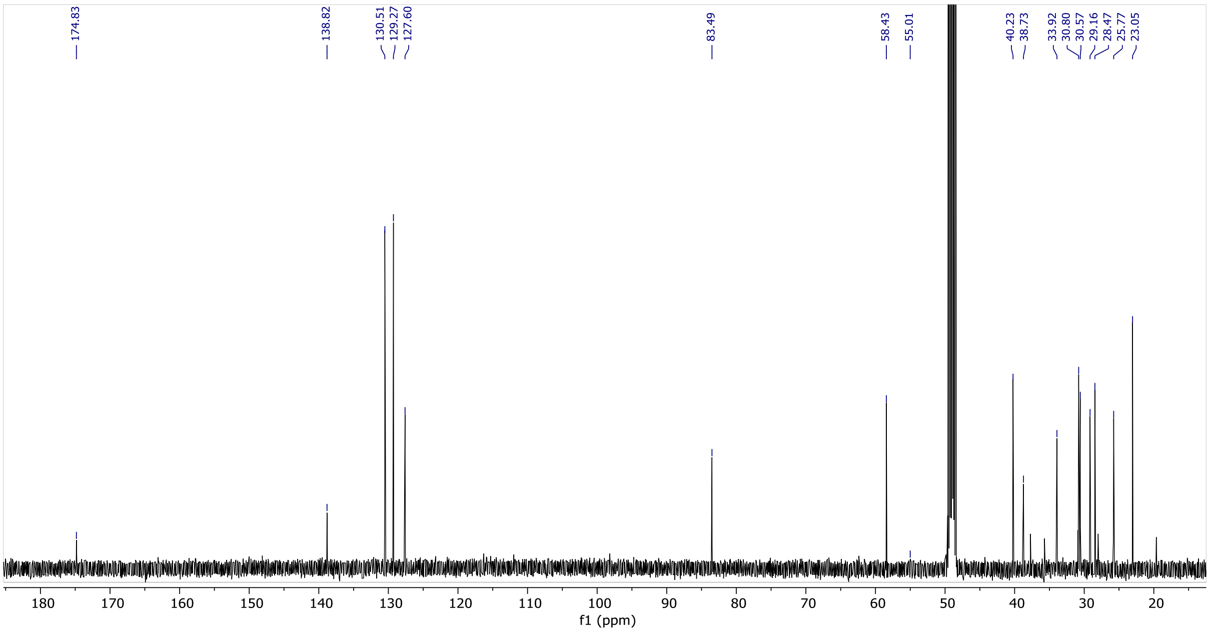

Supplementary Figure 10. (A) ^{1}H NMR (500 MHz) and (B) ^{13}C (125 MHz) spectra of P5

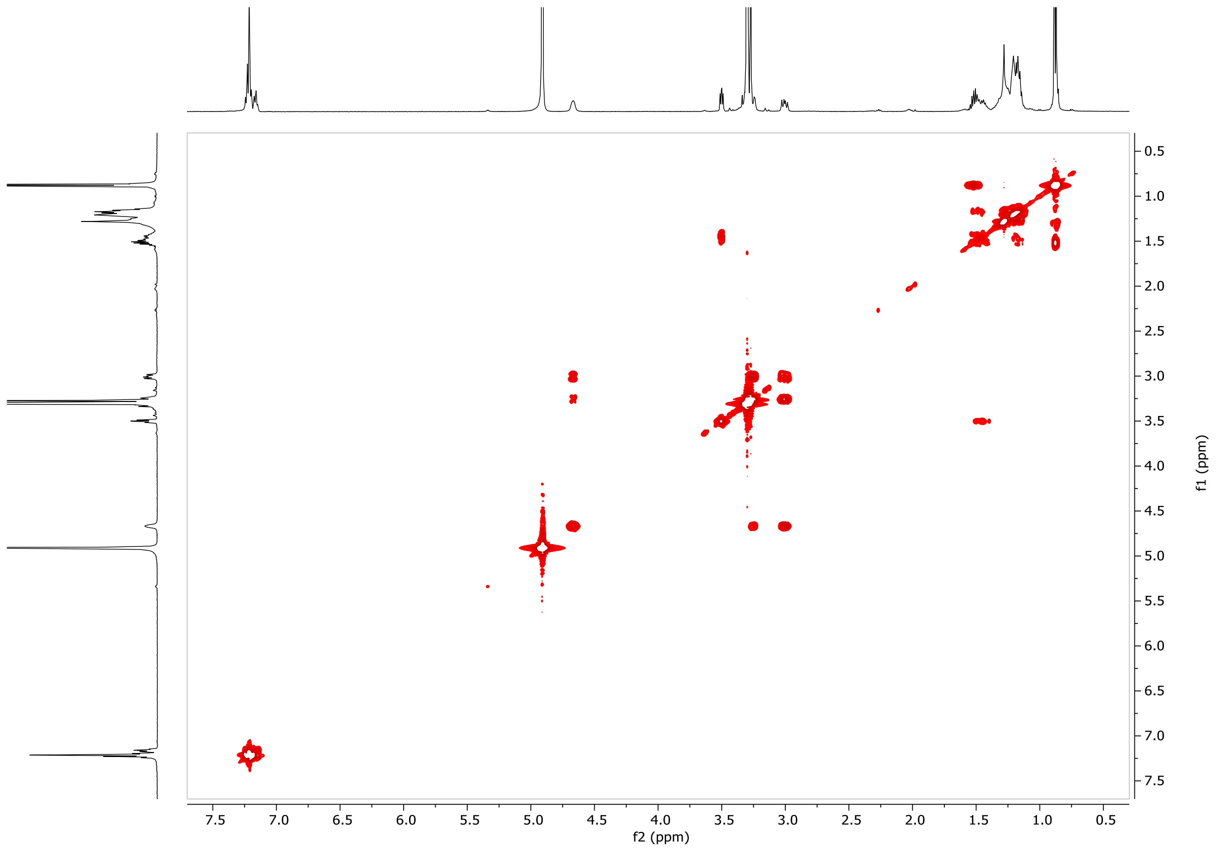

Supplementary Figure 11. COSY spectrum of P5 at 500 MHz

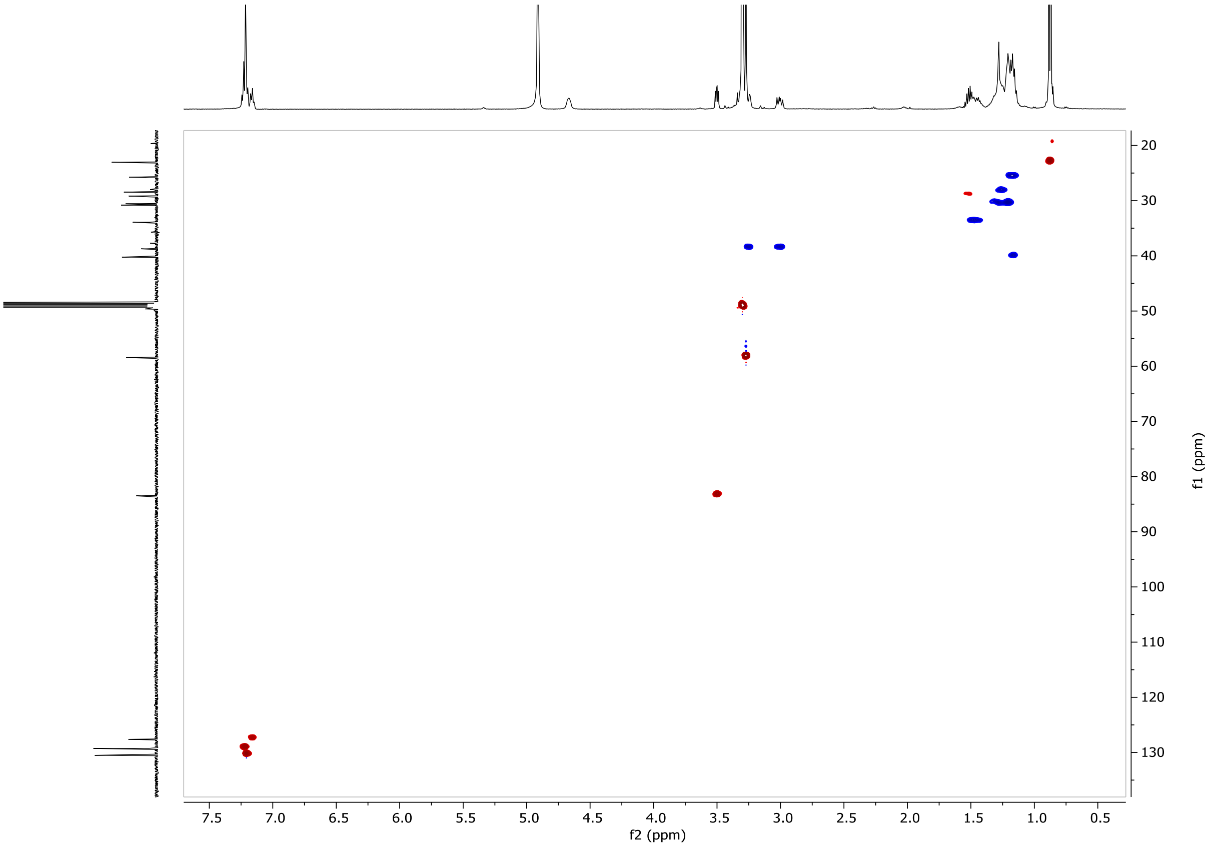

Supplementary Figure 12. HSQC spectrum of P5 at 500 MHz

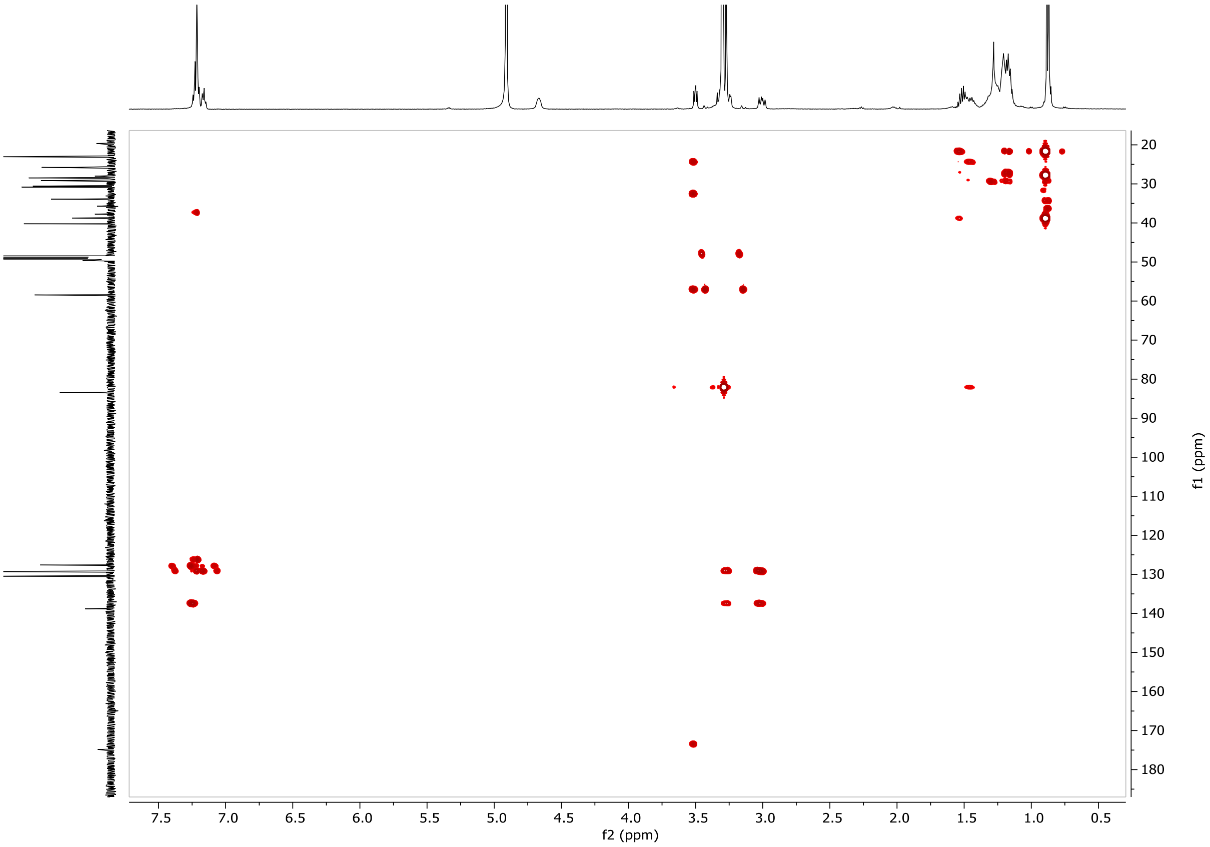

Supplementary Figure 13. HMBC spectrum of P5 at 500 MHz

Supplementary Table 1. Molecular docking analysis of P1-P4 with MDM2 (4HG7) and MDMX (3FE7)

Number	Compounds	Binding energy (kcal/mol)	
		MDM2 (4HG7)	Interacting amino acid residues
1	P1	-5.4	Ile19, Gln24, Lys51, Leu54, Phe55, Leu57, Gly58, Ile61, Phe86, Phe91, Val93, His96, Ile99, Tyr100
2	P2	-5.7	Ile19, Gln24, Lys51, Leu54, Phe55, Leu57, Gly58, Ile61, Phe91, Val93, His96, Ile99, Tyr100
3	P3	-5.8	Ile19, Gln24, Lys51, Leu54, Phe55, Leu57, Gly58, Ile61, Val75, Phe86, Phe91, Val93, His96, Ile99, Tyr100
4	P4	-5.7	Ile19, Gln24, Lys51, Leu54, Phe55, Leu57, Gly58, Ile61, Val75, Phe91, Val93, Ile99, His96, Tyr100

Number	Compounds	Binding energy (kcal/mol)	
		MDMX (3FE7)	Interacting amino acid residues
1	P1	-7.1	Met53, His54, Leu56, Gly57, Ile60, Met61, Tyr66, Gln71, His72, Val74, Phe90, Val92, Leu98
2	P2	-6.8	Met53, His54, Leu56, Gly57, Gln58, Ile60, Met61, Tyr66, Gln71, Val74, Phe90, Val92, Leu98
3	P3	-6.9	Met53, His54, Leu56, Gly57, Gln58, Ile60, Met61, Tyr66, Gln71, Val74, Phe90, Val92, Leu98
4	P4	-6.9	Met53, His54, Leu56, Gly57, Gln58, Ile60, Met61, Tyr66, Gln71, Val74, Phe90, Val92, Leu98

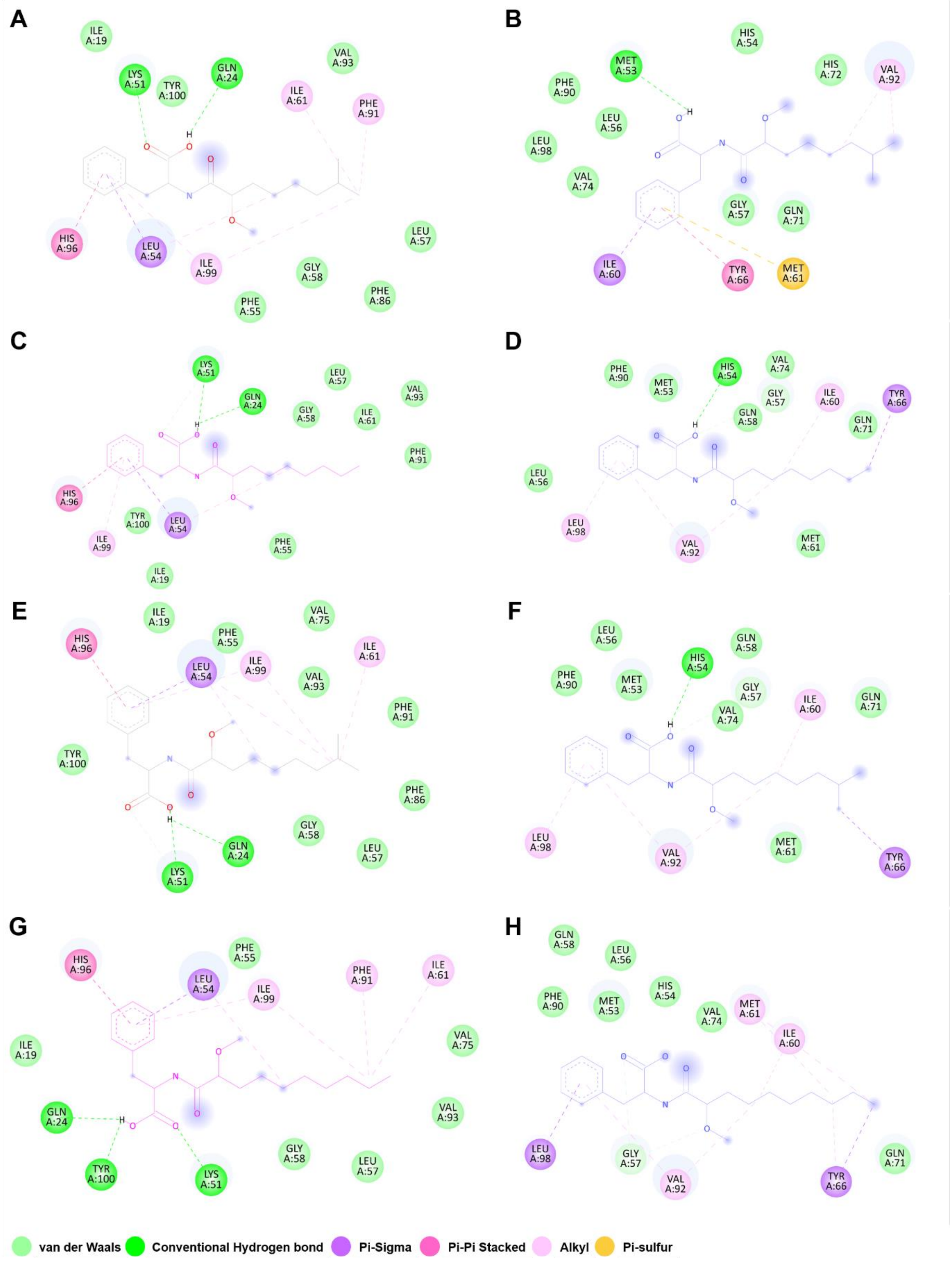

Supplementary Figure 14. 2D interaction diagram of P1(A, B), P2(C, D), P3(E, F), P4(G, H) with MDM2 (A, C, E, G) and MDMX (B, D, F, H), illustrating hydrogen bonding, π–π stacking, and hydrophobic interactions

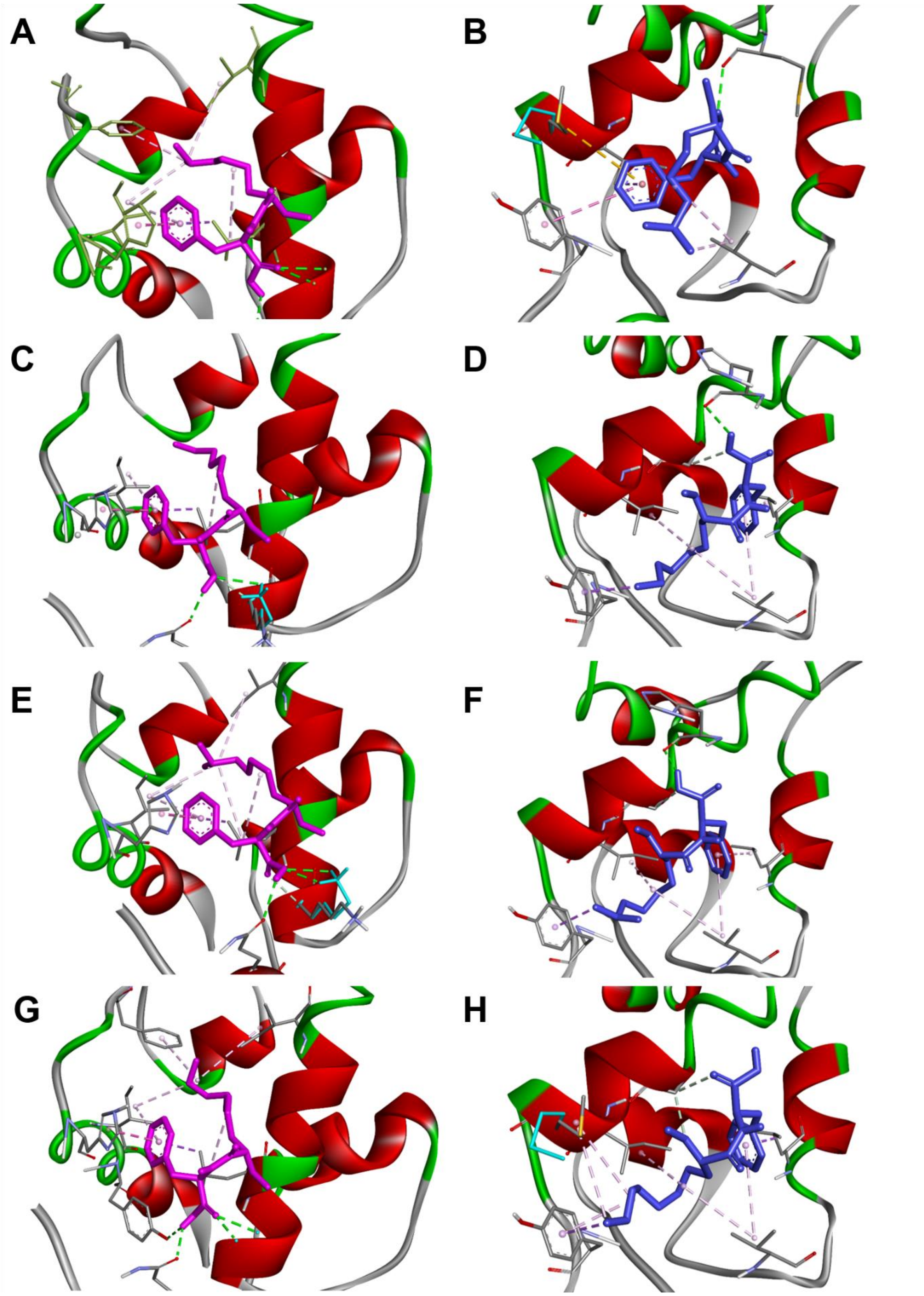

Supplementary Figure 15. 3D interaction diagram of P1(A, B), P2(C, D), P3(E, F), P4(G, H) with MDM2 (A, C, E, G) and MDMX (B, D, F, H)

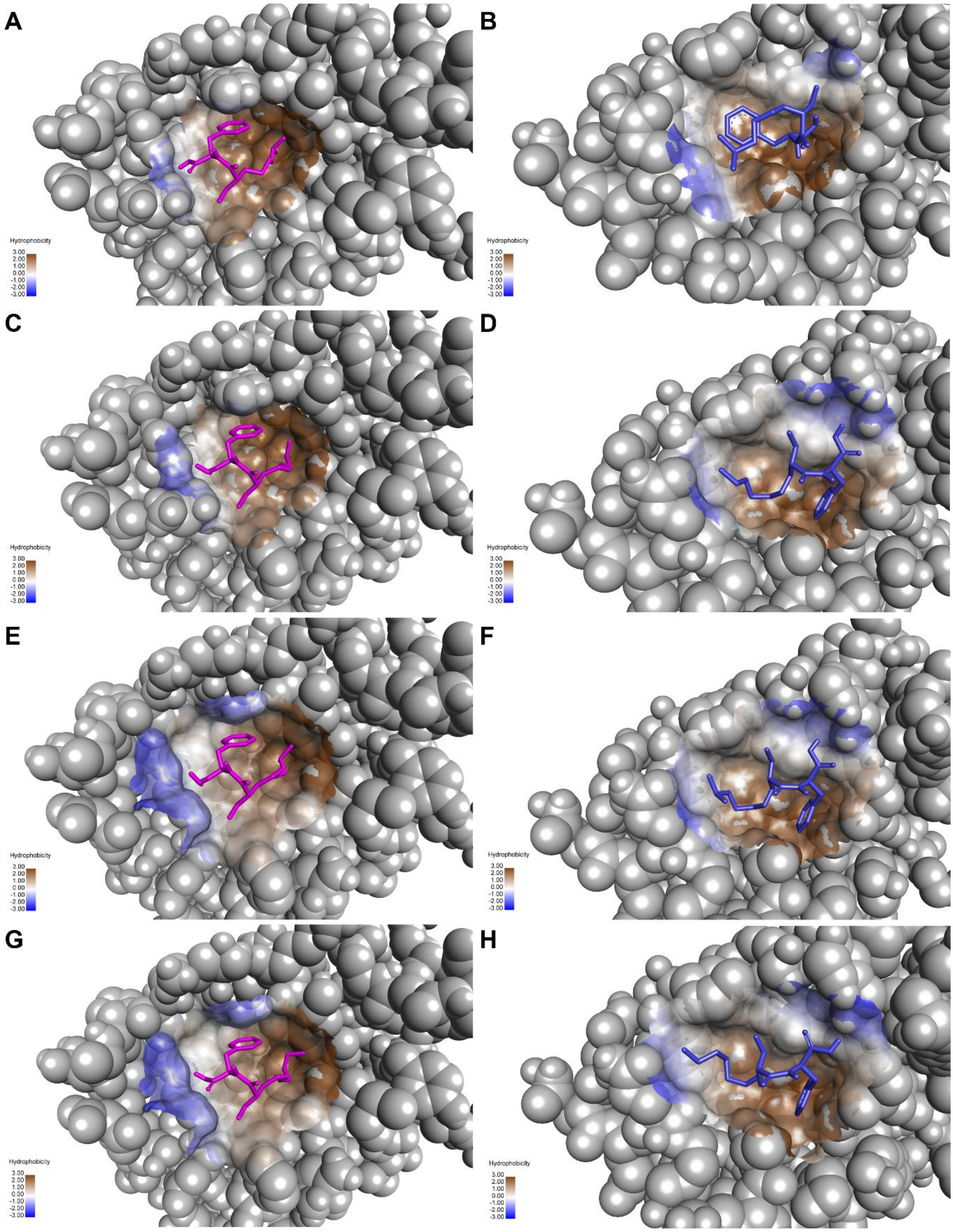

Supplementary Figure 16. Hydrophobic interaction surfaces of P1(A, B), P2(C, D), P3(E, F), P4(G, H) in complex with MDM2 (A, C, E, G) and MDMX (B, D, F, H)

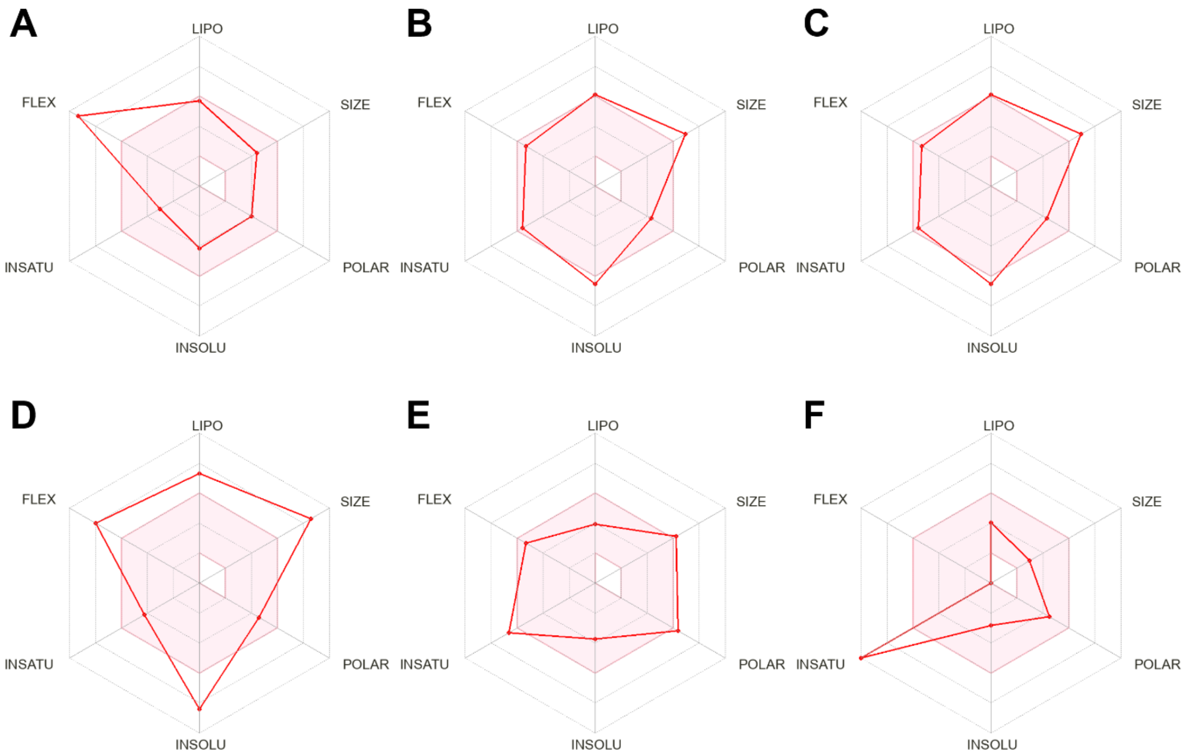

Supplementary Figure 17. Radar plots of six key ADME-related parameters predicted using SwissADME for P5 (A), Nutlin-3 (B), Nutlin-3a (C), RG-7112 (D), RO-5963 (E), and CTX1 (F). Each axis represents lipophilicity (LIPO), size (SIZE), polarity (POLAR), solubility (INSOLU), saturation (INSATU), and molecular flexibility (FLEX). The pink area indicates the optimal range for each properties
